# Time-Dependent Transcriptional Dynamics of Contextual Fear Memory Retrieval Reveals the Function of Dipeptidyl Peptidase 9 in Reconsolidation

**DOI:** 10.1007/s12264-024-01324-w

**Published:** 2024-12-02

**Authors:** Wen-Ting Guo, Wen-Xing Li, Yu-Chen Liu, Ya-Bo Zhao, Lin Xu, Qi-Xin Zhou

**Affiliations:** 1 Key Laboratory of Animal Models and Human Disease Mechanisms, Laboratory of Learning and Memory, Kunming Institute of Zoology, The Chinese Academy of Sciences, Kunming, 650223 China; 2 Kunming College of Life Science, University of Chinese Academy of Sciences, Kunming, 650204 China; 3 CAS Centre for Excellence in Brain Science and Intelligent Technology, Shanghai, 200031 China; 4 Jiangsu Key Laboratory of Neuropsychiatric Diseases, College of Pharmaceutical Sciences, Soochow University, Suzhou, 215123 China

**Keywords:** Transcriptome analysis, Temporal gene expression, Fear memory retrieval, Reconsolidation, DPP9

## Abstract

Numerous studies on the formation and consolidation of memory have shown that memory processes are characterized by phase-dependent and dynamic regulation. Memory retrieval, as the only representation of memory content and an active form of memory processing that induces memory reconsolidation, has attracted increasing attention in recent years. Although the molecular mechanisms specific to memory retrieval-induced reconsolidation have been gradually revealed, an understanding of the time-dependent regulatory mechanisms of this process is still lacking. In this study, we applied a transcriptome analysis of memory retrieval at different time points in the recent memory stage. Differential expression analysis and Short Time-series Expression Miner (STEM) depicting temporal gene expression patterns indicated that most differential gene expression occurred at 48 h, and the STEM cluster showing the greatest transcriptional upregulation at 48 h demonstrated the most significant difference. We then screened the differentially-expressed genes associated with that met the expression patterns of those cluster-identified genes that have been reported to be involved in learning and memory processes in addition to dipeptidyl peptidase 9 (DPP9). Further quantitative polymerase chain reaction verification and pharmacological intervention suggested that DPP9 is involved in 48-h fear memory retrieval and viral vector-mediated overexpression of DPP9 countered the 48-h retrieval-induced attenuation of fear memory. Taken together, our findings suggest that temporal gene expression patterns are induced by recent memory retrieval and provide hitherto undocumented evidence of the role of DPP9 in the retrieval-induced reconsolidation of fear memory.

**Supplementary Information:**

The online version contains supplementary material available at 10.1007/s12264-024-01324-w.

## Introduction

Memory retrieval, defined as “the access, selection, reactivation, or reconstruction of stored internal representations” [1], is a direct measure of memory. Increasing evidence implies that memory retrieval is an active and dynamic process. Once a memory is retrieved, it enters a labile state in which the original memory can be strengthened, weakened, or modified [2–5]. The destabilized memory relies on the mechanism of reconsolidation for its stabilization [5]. Repeated retrieval leads to the extinction of memory [3]. Whether retrieval enhances or erases memory is contingent upon multiple factors such as the strength and age of the memory, along with the duration of the retrieval cue [3, 6, 7]. Retrieval bidirectionally modulates memory and integrates new content into existing memory, promoting flexible survival strategies for individuals in a changing environment. Retrieval-induced destabilization provides opportunities for memory reprocessing and has emerged as a promising therapeutic target for psychiatric disorders and drug abuse in recent years [8–12].

To develop long-term stored memory, newly-learned information undergoes the process of consolidation. Memory consolidation involves cellular consolidation, which requires numerous transcription events and protein synthesis, as well as system consolidation, which involves long-term memory trace transfer from the hippocampus to the cerebral cortex [13–15]. Retrieval initiates the destabilization of established memory; therefore, transcription events and protein synthesis are also involved in reconsolidating the memory back into a stable state. It has been reported that the molecular mechanisms underlying consolidation and reconsolidation are largely shared. Studies in rodents have shown that *de novo* protein synthesis is not only essential for newly-learned information to be consolidated into stable long-term memories but is also needed for a retrieval-induced “labile” memory to be reconsolidated into a stable state [2, 16]. Transcription factors, including cAMP-response element-binding protein (CREB) [16], zif268 [17], and nuclear factor kappa B [5], as well as upstream protein kinases including extracellular signal-regulated kinase [18] and protein kinase A [19, 20], are required for consolidation and reconsolidation.

Several studies have shown that the molecular mechanisms underlying consolidation and reconsolidation do not always overlap, suggesting that they are distinct memory processes [21–23]. Moreover, studies have revealed that retrieval is accompanied by unique transcriptional signatures and an epigenomic landscape distinct from that of other memory phases [24–26]. Furthermore, some evidence has shown that retrieval-triggered processes, including memory modification, reconsolidation, and extinction have distinct biochemical signatures [3, 27, 28]. These findings suggest that memory retrieval leads memory to enter a dynamic state with high plasticity and that distinct molecular events mediate the direction in which labile memory is processed. The underlying synaptic plasticity mechanism of “reconsolidation-update” involves the dynamic exchange of glutamate-A2 (GluA2)-containing Ca^2+^-impermeable α-amino-3-hydroxy-5-methyl-4-isoaxolpropionic acid receptors (CI-AMPARs) and GluA2-lacking Ca^2+^-permeable AMPARs (CP-AMPARs) on synapses for a few hours following retrieval [29–31]. Retrieval-induced plasticity suggests that memory retrieval is not merely a recurrence of established synaptic plasticity from acquisition and consolidation, it is a dynamic process to modify memory contents, emphasizing the highly-regulated characteristics of memory reconsolidation-updating.

The molecular mechanisms of memory consolidation and retrieval are phase-dependent processes [32–34]. Given that reconsolidation depends on both the consolidation and retrieval of memory, the question of whether the molecular events induced by retrieval change with time is raised. Some reports imply that there are differences in regulatory mechanisms between recent and remote memory reconsolidation [3, 20] and that retrieval-induced outcomes vary over time [35]. Elucidating the phase-dependent regulatory mechanism of the memory retrieval-induced process will aid in further understanding the function of retrieval-induced memory reprocessing, and identifying molecular markers that can be used to estimate memory status (reconsolidation or extinction) will help identify possible therapeutic targets for psychiatric disorders.

To investigate the dynamic molecular mechanisms of memory retrieval-induced memory reprocessing during the recent phase, we focused on the hippocampus-dependent recent contextual memory retrieval process. We applied a transcriptome analysis of hippocampal tissue from rats that underwent contextual fear memory retrieval at 0.5, 24, 48, and 72 h. We identified the most strongly expressed cluster, which presented the highest gene expression level at 48 h. Further screening of differentially-expressed genes (DEGs) that followed the expression trend of this cluster led to the identification of nine candidate genes relevant to retrieval at the 48-h time point. The results of the behavioral qPCR validation suggested that these candidate genes were induced by 48 h of memory retrieval. We specifically focused on a novel candidate gene, dipeptidyl peptidase 9 (*DPP9*), which had not been previously explored in the context of learning and memory. Subsequent animal experiments revealed its involvement in memory retrieval and reconsolidation at 48 h after learning. Our findings suggest that temporally dynamic molecular events are induced by recent memory retrieval and introduce DPP9 as a novel regulator of memory reconsolidation.

## Materials and Methods

### Animals

Male Sprague‒Dawley rats (from Vital River Laboratory Animal Technology Co. Ltd., Beijing, China), aged 10‒12 weeks were housed in groups in ventilated cages with wood chip bedding and maintained on a 12-h light-dark cycle. They had free access to food and water and lived in a constant temperature environment. Each rat was handled for ~2 min per day for 3 consecutive days prior to the behavioral experiment. All procedures were approved by the Animal Ethics Committee of Kunming Institute of Zoology, Chinese Academy of Sciences (Approval Number: SMKX-20171118-158).

### Contextual Fear Conditioning

The contextual fear conditioning procedures were adapted from previously described methods [36–38]. Behavioral experiments were conducted using equipment from MED Associates Inc., Vermont (USA). On the day of fear conditioning, each rat was given 2 min to freely explore the context before receiving 6 foot-shocks (0.8 mA for 2 s) with an average interval of 90 s between shocks (i.e., 6 trials). Two minutes after the last trial, the rat was returned to its home cage. The retrieval tests were subsequently applied in the same chamber at 0.5, 24, 48, or 72 h after training, each lasting 5 min. Freezing levels were evaluated *via* an automated scoring system (Med Associates Inc.).

### Sample Preparation and Transcriptomic Assays

Each rat was sacrificed 1 h after retrieval, and the intact hippocampus was isolated. The dissected samples were incubated in RNAlater® solution (Ambion, USA) at room temperature for 30 min and then stored at − 80 °C. The RNA concentration was quantified with a Qubit® RNA HS Assay Kit and a Qubit® 2.0 Fluorometer (Life Technologies, USA), and RNA integrity was assessed on a Bioanalyzer 2100 system (Agilent Technologies, USA). One microgram of total RNA from each sample with RNA integrity number (RIN) values > 8 was used. The paired-end sequencing library was constructed using the NEBNext® Ultra II Directional RNA^TM^ Library Prep Kit (New England Biolabs, USA), and an index code was assigned to each sample. mRNA was purified from total RNA using poly-T oligo-attached magnetic beads and then cleaved into small pieces by divalent cations in 5× first-strand cDNA synthesis reaction buffer (Invitrogen, USA) at high temperature. The RNA fragments were reverse transcribed into first-strand cDNAs *via* SuperScript® II reverse transcriptase (Invitrogen, USA) with random primers. The second-strand cDNAs were subsequently synthesized with DNA polymerase I and RNase H. These cDNA ends underwent a transformation process to convert them into flat ends, and a single “A” base was added, which was followed by ligation of the indexing adapters. The products were purified and then amplified by the polymerase chain reaction (PCR). These PCR products were then purified on the AMPure XP system (Beckman Coulter, USA) to create the final cDNA library. The sequencing library was quantified with the KAPA Library Quantification Kit-Illumina® Platforms and with the Agilent Bioanalyzer 2100 system (Agilent Technologies). Indexed libraries were clustered by the TruSeq PE Cluster Kit v3-cBot-HS (Illumina, USA) on a cBot cluster amplification system. The clustered cDNA libraries were then submitted to an Illumina NovaSeq (Illumina), and paired-end (2 × 150 bp) reads were produced.

### RNA-Seq Raw Data Processing and Gene Annotation

Raw data processing was based on the procedure developed by Mihaela et al. [39, 40]. Low-quality reads were deleted by Skewer (v0.2.2). Unpaired reads of FASTQ files were trimmed by Pairfq (v0.17.2). The clean paired-end reads were aligned against the rat reference genome based on gene annotation data from the Ensemble database (mRatBN7.2) via HISAT2 (v2.0.5) [41]. The transcriptomes were assembled, and the transcript expression levels were quantified through StringTie (v1.3.3b) [42] and then presented as fragments per kilobase of transcript sequence per million mapped reads (FPKM) through the Ballgown software package (v2.2.0) [43].

Rat gene annotation files were obtained from the ensemble database (http://www.ensembl.org/index.html) using the BioMart tool. The annotated file contains the following information: gene stable ID, gene type, gene name, chromosome/scaffold name, gene start, gene end, gene description, and gene % GC content. The RNA-sequencing (RNA-seq) files were subsequently annotated using a self-programmed R script. There were 21 types of RNA in the annotated expression matrix, and the protein-coding mRNAs were extracted from the annotated files for further analysis. To ensure data quality, genes with a log_2_ (FPKM) > 0.1 for every sample in each group were selected for analysis.

### Differential-Expression Analysis

DEGs were identified using R Statistical Software (v3.4.1). The empirical Bayes algorithm of the limma package (version 3.30.13) [44] was applied to analyze genes differentially-expression between samples from different groups. Fold change (FC) > 1.2 and < 0.83 (the reciprocal of 1.2) with a corrected false discovery rate *P* value < 0.05 were used as the thresholds for the identification of the upregulation and downregulation of DEGs between selected groups. Rats without any behavioral experience composed the control group. The differential expression between the retrieval groups at different time points and the control group was analyzed. A heatmap of DEGs in different groups was drawn with a heatmap package, and hierarchical clustering was performed with the ward. D2 algorithm.

### Short Time-Series Expression Miner

Temporal gene expression profiles of memory retrieval were generated by using the short time-series expression miner (STEM) tool [45]. The experimental grouping was the same as that for the above DEG analysis. The control group included rats without any behavioral experience, and the data from the four-time point retrieval groups, including those at 0.5, 24, 48, and 72 h after training, were collected for STEM analysis. The input parameters included the logFC of the four time-point retrieval groups compared with the control group, with the other parameters set to default values. With the STEM clustering method [46], all genes were classified into 50 categories based on their expression trends, and the expected number of genes in each category was calculated. Categories were subsequently fitted and merged to illustrate the overall characteristics of logFC over time. The *P*-value was computed by assessing the difference between the expected and actual gene numbers. A *P*-value < 0.05 was considered statistically significant.

### Screening of the STEM Cluster Trend DEGs

Based on the gene clusters derived from the STEM analysis, we designed the following method to screen the DEGs that met a specific trend:

*Step 1* The DEGs and their FCs at different time points compared with those in the control group were calculated.

*Step 2* Genes that were consistently dysregulated at all time points were screened. Let $G$ be the set of DEGs, where the subscripts correspond to DEGs at different times and where $G_{con.}$ Indicates a gene with consistent differential expression:

$$ G_{con.} = G_{0.5 h} \cap G_{24 h} \cap G_{48 h} \cap G_{72 h} $$

*Step 3* Genes matching specific expression trends were screened. Let $logFC$ be the FC of a gene, and the subscripts correspond to different times. Let $G_{final}$ be the final screened genes, and for each gene $g$, the screening conditions are as follows:

$$ \forall g \in G_{final} , logFC_{{g_{48 h} }} > \left( {logFC_{{g_{24 h} }} ,logFC_{{g_{72 h} }} } \right) > logFC_{{g_{0.5 h} }} $$

*Step 4* The $logFC$ values of all the genes are finally normalized after the screening:

$$ logFC_{g} = logFC_{g} - logFC_{{g_{0.5 h} }} $$

### Gene Set Enrichment Analysis and Pathview Analysis

Pathway gene set information for the rats was obtained from the Kyoto Encyclopedia of Genes and Genomes (KEGG) database, while biological process information was sourced from the QuickGO database. All the gene set files were subsequently formatted, and gene set enrichment analysis (GSEA Java desktop application, v3.0) of the four comparison groups was applied. Gene sets with < 10 or > 500 genes were excluded from analysis. The mean t-statistic of genes was computed within each KEGG pathway and Gene Ontology (GO) using permutations with 1000 repetitions. The upregulated pathways were characterized as those with a normalized enrichment score (NES) > 0, whereas the downregulated pathways were defined as those with an NES < 0. The identification of significantly enriched pathways and GO terms was based on a false discovery rate P value < 0.05. Visual analysis of the significantly enriched KEGG pathways was carried out using the path view package [47]. The FCs of all the genes were used as input parameters, and the other parameters were set to the defaults.

### Gene Function Interaction Network Analysis

The functional interaction network of the identified and related genes was constructed on the GeneMANIA web server (http://genemania.org/) [48]. The network was generated based on their relationships, such as coexpression, colocalization, pathway, physical interaction, genetic interaction, shared protein domain, and prediction. The biological processes associated with these genes during GO analysis were automatically generated.

### Protein–Protein Docking

The crystal structures of GluA2 and DPP9 were downloaded from the Protein Data Bank (PDB) database (https://www.rcsb.org/), with the identifiers 6DM1 and 6EOQ. The GluA2 complex contains 3 domains: an amino-terminal domain (ATD), a ligand-binding domain (LBD), and a transmembrane domain (TMD) [49]. The GRAMM docking web server (https://gramm.compbio.ku.edu/) was used to perform protein docking of GluA2 and DPP9. Owing to the high molecular weight of the GluA2 complex, the LBD + TMD domains and the TMD domain of GluA2 were selected for protein docking with DPP9. The generated results of each protein docking pair included 10 models. All of these models were visualized with Discovery Studio 2016.

### Real-Time Quantitative Polymerase Chain Reaction in RNAlater®

Total RNA was extracted from hippocampal tissue for transcription, and cDNA was generated by the PrimeScript Reverse Transcriptase Reagent Kit (Takara, Japan). qPCR was performed with a hot-start SYBR Green mix kit (Takara, Japan), and an Applied Biosystems 7500 Real-Time PCR System (Applied Biosystems, USA) was used in the experiments. The reactions were performed in a volume of 20 μL according to the manufacturer’s instructions. Relative mRNA expression levels were calculated by the comparative CT method. The sequences of the primers (Sangon Biotech, China) used for real-time PCR are shown in Table 1.

**Table 1:** Primers used for quantitative real-time polymerase chain reaction (RT-qPCR).

Gene		Primers (5′–3′)
Adora1	Forward	CCTGAGTGTGGTAGAGCAAGA
	Reverse	CCAGACGAAGAAGTTGAAGTAGAC
CCND2	Forward	TACCTCCCGCAATGTTCCTAT
	Reverse	GTTCCTCACAGACCTCTAGCA
Dlg4	Forward	AGATCCTGGCGGTCAACA
	Reverse	AGGTGCTGAGAATACGAGGTT
DPP9	Forward	TGGTAGCCGCAAGTCATC
	Reverse	CATAAGGCATTCCCAGGTAATAGA
GNB4	Forward	TCACAGGACACATCTCAGACAT
	Reverse	CAGGTGGCGTCATCAGAAC
PI3KCA	Forward	GGTGGAATGAATGGCTGAATTATG
	Reverse	CGGACAGTGCTCCTCCTTAG
Shank3	Forward	GTTCCTATGACAGCCTCACTTC
	Reverse	GCCACACCTTCCACATCTAC
Zif268	Forward	CACCTGACCACAGAGTCCTTT
	Reverse	ATGAAGAGGTTGGAGGGTTGG
Arc	Forward	GTGAATGACACCAGGTCTCAAG
	Reverse	GGCACCTCCTCTTTGTAATCC
GAPDH	Forward	CATGGCCTTCCGTGTTCCTA
	Reverse	TACTTGGCAGGTTTCTCCAGG
Npas4	Forward	GCTTCCGTGTCACTGATAGG
	Reverse	ACGCTTCTTCTCAACACTACC

### Protein Extraction and Western Blotting

After all of the behavioral experiments, the hippocampal GFP-positive tissue was extracted in precooled 1× phosphate-buffered saline (PBS). The samples were lysed in RIPA lysis buffer (Solarbio, China) containing protease inhibitor (Millipore, USA). The protein concentrations were determined with a BCA Protein Assay Kit (Solarbio, China). Approximately 60 μg protein samples were separated on 7.5% SDS‒PAGE gels and transferred to PVDF membranes (Millipore, USA). The blots were blocked with 5% BSA for 1 h at room temperature and then incubated with a mouse anti-DPP9 antibody (OriGene Technologies, USA) overnight at 4°C. A rabbit anti-GAPDH antibody (Abcam, USA) was used to normalize the amount of sample loaded. The membranes were subsequently washed with TBST and incubated a with goat-anti-mouse HRP-conjugated secondary antibody or goat-anti-rabbit HRP-conjugated secondary antibody (Abcam, USA) for 1 h at room temperature. The membranes were washed again with TBST, and the signals were visualized with a chemiluminescent substrate reagent kit (Thermo Fisher Scientific, USA). Relative protein intensities were calculated in Quantity One software (Bio-Rad, USA) after normalization to the GAPDH signal.

### Surgery and Cannula Implantation

The rats were anesthetized by intraperitoneal injection of pentobarbital sodium (Sigma, 60 mg/kg). Medical oxygen (95% O_2_ and 5% CO_2_) was delivered through a mask, and the body temperature (37 ± 0.5°C) was maintained by an animal electric blanket until the rats woke from anesthesia. The operation was performed on a stereotaxic apparatus (RWD Life Sciences, China). Stainless steel guide cannulas (26 G) were implanted into the CA1 regions of the dorsal hippocampus (dCA1) at the following coordinates: anteroposterior (AP), − 3.8 mm; mediolateral (ML) ±2.8 mm; and dorsoventral (DV) − 2.6 mm according to the Rat Brain Atlas (sixth edition) [50]. Guide cannulas were implanted 1 mm above the target region because the injection needles were 1 mm longer. The guide cannulas were affixed to the skull of the rat with dental cement, and three stainless steel screws were used as anchors. The guide cannulas were plugged with stylets after they were secured to the skull.

### Drug Infusions

Drugs were infused at least one week after surgery. Val-boroPro (VBP) (MedChemExpress, USA), a non-selective inhibitor of post-proline cleaving serine proteases, including DPP9 [51–53], was diluted to 100 μmol/L with saline infusion. The rats received bilateral infusions (1.5 μL/side) of either VBP or saline through a Hamilton syringe operated by a microinfusion pump (RWD Life Sciences, China). The infusions were administered at 0.5 μL/min, and the injectors were left in place for 5 min following infusion to ensure sufficient diffusion of the drug or vehicle**.** Bilateral infusions were administered simultaneously via a dual-syringe micropump. During infusion, the rats were wrapped in a towel and temporarily restrained in the hands of the operator. After infusion, the rats were returned to their home cages and left for 1.5 h before the behavioral experiment began.

### Stereotaxic Virus Delivery

The virus AAV2/9-hSyn-dpp9-3xFLAG-WPRE-pA and the virus AAV2/9-hSyn-GFP-WPRE-pA were from OBIO Technology (China). For the DPP9 overexpression group, both AAV2/9-dpp9-3xFLAG-WPRE-pA and AAV2/9-hSyn-GFP-WPRE-pA mixed viruses were injected. For the control group, the AAV2/9-hSyn-GFP-WPRE-pA virus was injected. The virus was injected into dCA1 (in mm: AP, − 3.8, ML ± 2.8, and DV − 2.6) and driven through a glass micropipette by a syringe pump at 0.1 μL/min. The total injection volume per side of the dCA1 region was 1 μL. The micropipette was left in place for 5 min and pulled out slowly. The scalp of each rat was sutured and disinfected with iodophor.

### Statistical Analysis

All values are presented as the mean ± SEM. One-way analysis of variance (ANOVA) was used to compare the mRNA expression levels of specific genes at different time points during retrieval. The learning curve was analyzed by two-way ANOVA (Figs 3B, C left, D, 5B, and 6B, C). The freezing responses associated with contextual fear memory retrieval were analyzed by one-way ANOVA (Figs 3B, C right, 5C). Statistical analysis was applied using GraphPad Prism 8.3. A *P* value < 0.05 was considered significantly different.

## Results

### Differential Gene Expression and Gene Set Enrichment Analysis of Memory Retrieval at Different Time Points

**Fig. 1:**
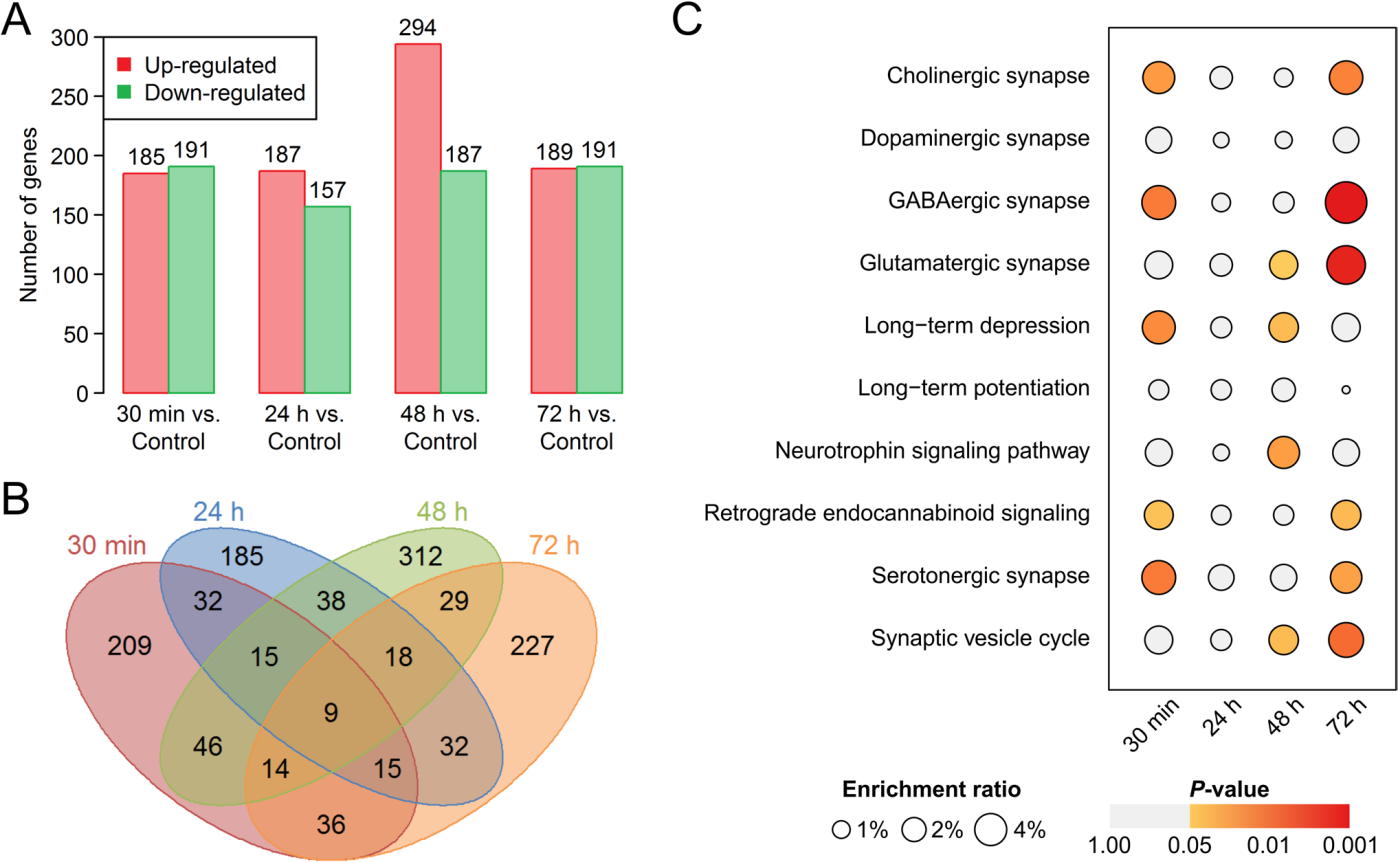
Numbers of differentially-expressed genes (DEGs) and enriched KEGG pathways associated with synaptic functions at various time points of memory retrieval. **A** Numbers of upregulated and downregulated genes in the 0.5, 24, 48, and 72 h post-learning retrieval groups compared with the control group. **B** Venn diagram of DEGs in different time point retrieval groups compared with the control group. **C** Synaptic function-related pathway enrichment of DEGs in different time point retrieval groups. The enrichment ratio is calculated as the number of DEGs divided by the total number of genes in the pathway. A *P*-value < 0.05 was considered significant. (Each group *n =* 3; all gene expression values are standardized by Z-score).

During the investigation of memory retrieval, the rats underwent contextual fear conditioning, and fear memory was subsequently retrieved at 0.5, 24, 48, and 72 h after learning. To assess transcriptomic alterations induced by different stages of memory retrieval, the rats were sacrificed 1 h after retrieval, and hippocampal tissue was collected for transcriptome analysis. The analysis of DEGs revealed distinctive patterns across the time points. In the 0.5 h retrieval group, 185 genes were upregulated, and 191 were downregulated compared with those in the control group. Similarly, the 24-h retrieval group included 187 upregulated and 157 downregulated genes, the 48-h retrieval group included 294 upregulated and 187 downregulated genes, and the 72-h retrieval group included 189 upregulated and 191 downregulated genes (Fig. 1A). A considerable number of these genes were expressed at higher levels in the 48-h retrieval group than in the other groups (Fig. S1). A Venn diagram revealed that the majority of specific DEGs were expressed at 48 h (Fig. 1B). KEGG analysis revealed that distinct pathways were significantly enriched in the different comparison groups (Fig. S2A). Notably, the 0.5 h retrieval group presented the most significant changes in the number of pathways. In the 48 h retrieval group, pathways such as lysine degradation and glutamatergic synapses were significantly upregulated. Conversely, pathways related to long-term depression, cGMP-PKG signaling, Notch signaling, aminoacyl-tRNA biosynthesis, amino sugar, and nucleotide sugar metabolism, and the cell cycle were notably downregulated in the 48 h retrieval group (Fig. S2A). Visualization of the KEGG pathways is shown in Fig. S2B. Further exploration of synaptic function-related pathways through KEGG analysis revealed that DEGs from different retrieval time points were enriched in distinct pathways, underscoring differences in synaptic plasticity among them (Fig. 1C). Notably, the 48-h retrieval group exhibited significant enrichment of pathways associated with glutamatergic synapses, long-term depression, neurotrophin signaling, and the synaptic vesicle cycle (Fig. 1C).

### STEM Analysis Identifies a High Expression Cluster at 48 h Retrieval After Contextual Fear Conditioning

**Fig. 2:**
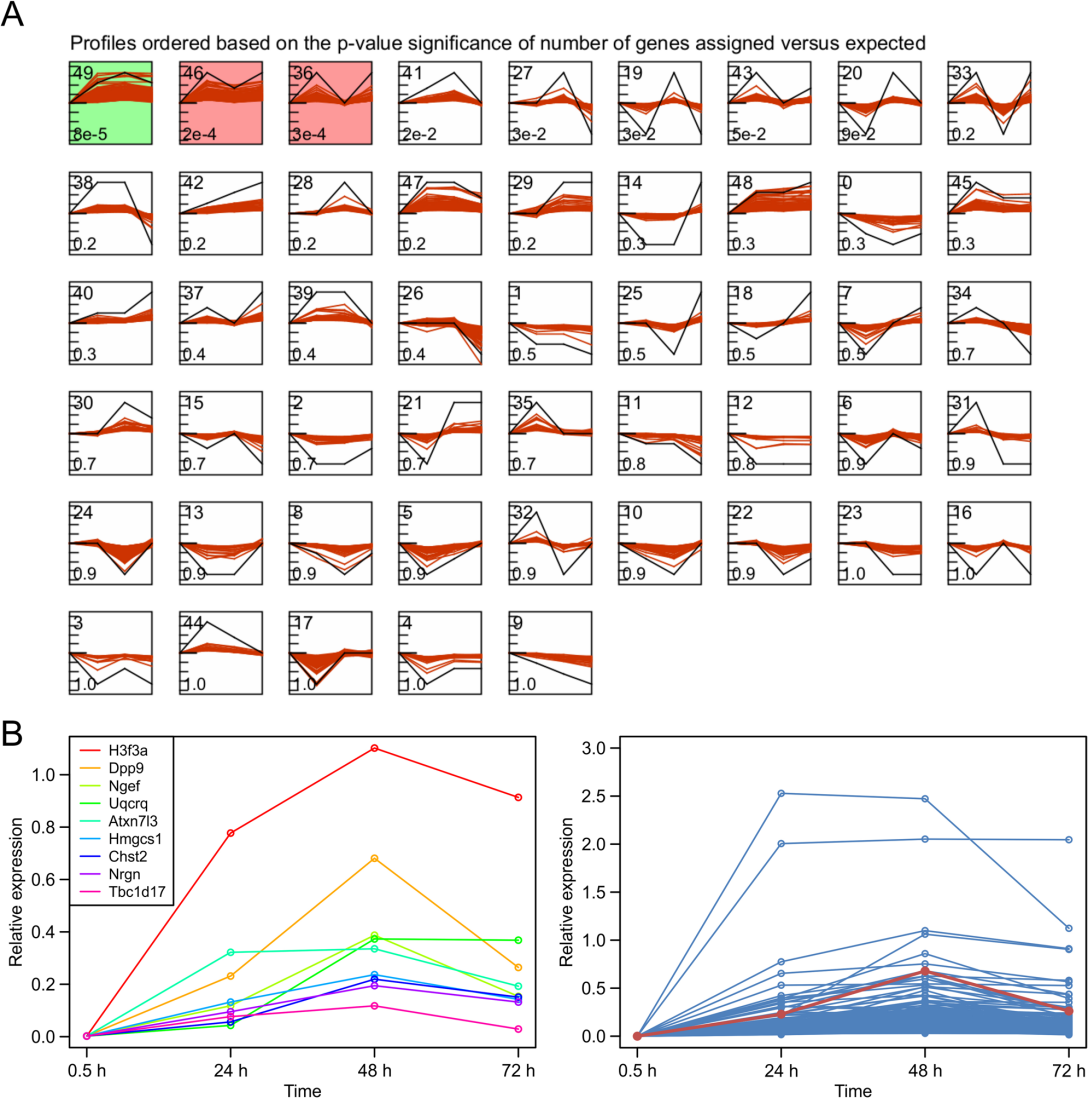
STEM analysis of temporal gene expression profiles during fear memory retrieval at different time points. **A** List of all STEM analysis results. Red line, the expression trend of each gene; black line, the overall expression trend of each cluster. Time points are indicated at equal intervals. The cluster number is displayed on the upper left of the square frame. Significantly enriched clusters are distinguished by the background color of the frame (*P* < 0.05). **B** Left, summary of the expression trends of cluster 49 characteristic DEGs; right, summary of all cluster 49 genes and cluster 49 characteristic DEGs. Red line, the expression trend of DPP9.

STEM analysis revealed three clusters with significant assignments, namely clusters 49, 46, and 36 (Figs 2A and Fig. S3). The baseline for each cluster was established by the logFC of genes in the 0.5 h retrieval group *versus* the control group. In cluster 49, gene expression levels at 24, 48, and 72 h were notably upregulated, particularly at 48 h. Clusters 46 and 36 presented peak gene expression levels at 24 h and 72 h, respectively. Given that the 48-h retrieval group presented the greatest number of DEGs and significant upregulation of synaptic function-related pathways, our primary focus was on genes highly expressed at 48 h. The algorithm identified 9 DEGs in cluster 49: *H3f3a*, *Dpp9*, *Ngef*, *Uqcrq*, *Atxn7l3*, *Hmgcs1*, *Chst2*, *Nrgn*, and *Tbc1d17* (Fig. 2B left). Notably, most of these genes are known to be associated with learning and memory processes. The expression curves of all screened genes aligning with the trend of cluster 49 are summarized on the right side of Fig. 2B. Additional details on the genes in cluster 49 can be found in Table S1, and the GO enrichment results of cluster 49 are listed in Table S2.

### QPCR Verification of Highly-Expressed Genes Induced by 48 h Fear Memory Retrieval

**Fig. 3:**
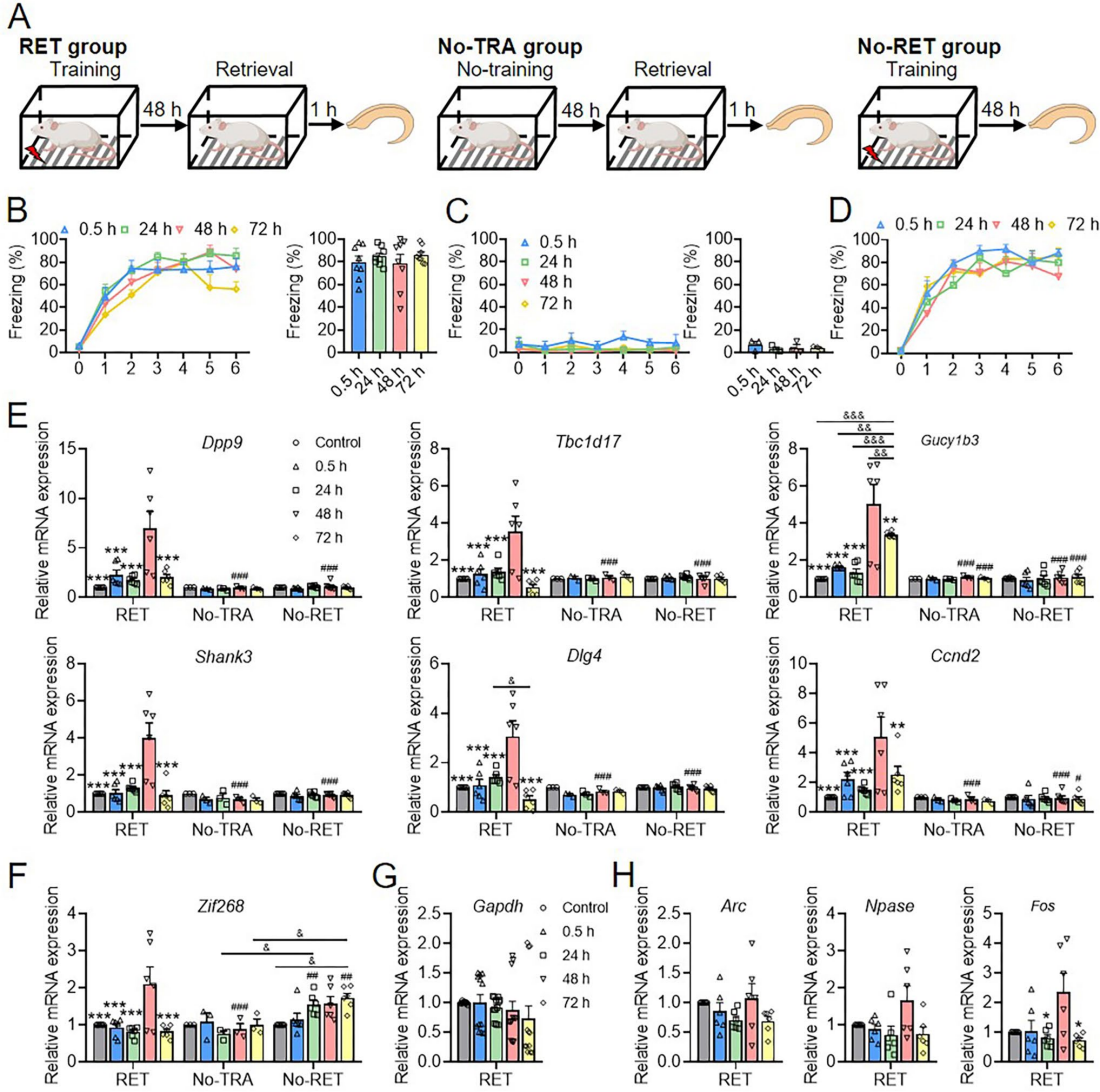
QPCR verification of transcriptome results. **A** Diagram of three behavior designs for qPCR verification. **B-D** The learning curves and memory retrieval performance of RET (*n =* 8 per time point group), No-RET (*n =* 3 per time point group), and No-TRA group (*n =* 6 per time point group). **E** QPCR verification of genes: *Dpp9*, *Tbc1d17*, *Gucy1b3*, *Shank3*, *Dlg4,* and *Ccnd2*. (*n =* 6 for all RET group, *n =* 3 for all No-TRA group, *n =* 6 for all No-RET group). **F** QPCR result of *Zif268* (*n* value is the same as above). **G** QPCR result of *Gapdh*.** H** QPCR results of *Arc* (*n =* 6), *Npase4* (*n =* 6), and *Fos* (*n =* 6). For learning curve and q-PCR results (**E**, **F**) statistical comparisons, two-way RM ANOVA followed by Bonferroni’s post-tests; for memory retrieval and q-PCR results (**G**,** H**) statistical comparisons, one-way ANOVA followed by Bonferroni’s post-tests. *P < 0.05, **P < 0.01, ***P < 0.005, *contrast effects for control, 0.5, 24, and 72 h *versus* 48 h in the RET group; ^#^*P* < 0.05, ^##^*P* < 0.01, ^###^*P* < 0.005, ^#^contrast effects for a certain time point of No-TRA or No-RET group *versus* the same time point of the RET group; ^&^*P* < 0.05, ^&^contrast effects for comparison as shown in the line. Values are the mean ± SEM.

To validate whether the molecular events identified in the transcriptome results were indeed induced by memory retrieval, we implemented three behavioral designs for qPCR confirmation: the training and retrieval (RET) group, the training but no retrieval (No-RET) group, and the pseudotraining (no shock) and retrieval (No-TRA) group (Fig. 3A). The learning curve and retrieval performance of different time point retrieval groups in the RET behavior design demonstrated a substantial fear memory level (Fig. 3B). Conversely, the learning curve and retrieval performance of the No-RET groups indicated almost no fear memory (Fig. 3C). In the No-RET design, the learning curves of the different time point retrieval groups revealed satisfactory performance in fear memory acquisition (Fig. 3D). The genes selected for qPCR verification included cluster 49 characteristic DEGs (*Dpp9* and *Tbc1d17*), as well as *Gucy1b3* and *Shank3* in the cGMP‒PKG signaling pathway, *Dlg4* in glutamatergic synapse pathways, and *Ccnd2* in prolactin pathways, all of which were upregulated at 48 h (Fig. S2B). The results of qPCR revealed significant upregulation of the verified genes at 48 h in the RET group but not in the No-RET and No-TRA groups (Fig. 3E). *Zif268* was also chosen for the behavioral qPCR verification experiment, revealing that elevated *Zif26*8 expression was closely associated with learning and 48 h fear memory retrieval, as evidenced by the significantly elevated level of *Zif268* expression in the No-RET and RET groups at the 48 h time point (Fig. 3F). There was no notable change in the expression of the housekeeping gene *Gapdh* (Fig. 3G). Furthermore, additional immediate-early genes such as *Arc*, *Npase4*, and *Fos* were detected in the RET behavioral design. Among these genes, only *Fos* displayed an elevated expression level at 48 h (Fig. 3H), which aligns with the upregulated *Fos* expression in the osteoclast differentiation pathway at 48 h (Fig. S2B). These results suggest that cluster 49 characteristic DEGs and genes significantly upregulated at the 48 h time point in enriched KEGG pathways were induced by the retrieval of fear memory at 48 h. In addition, the qPCR results of immediate-early genes suggested that certain transcription factors, such as *Zif268* and *Fos*, may be involved in the time-dependent dynamic regulatory mechanism of the memory retrieval-induced process [54, 55].

### DPP9 Functional Prediction and Docking Simulation with GluA2

Dipeptidyl peptidase 9, an N-terminal post-proline cleaving enzyme belonging to the DPPIV family, has been extensively studied in the fields of metabolism [56, 57], oncology [58–60], inflammation, and immunity [61–63]. Despite its abundant expression in the brain, its function in the central nervous system has remained unexplored [64–66].

**Fig. 4:**
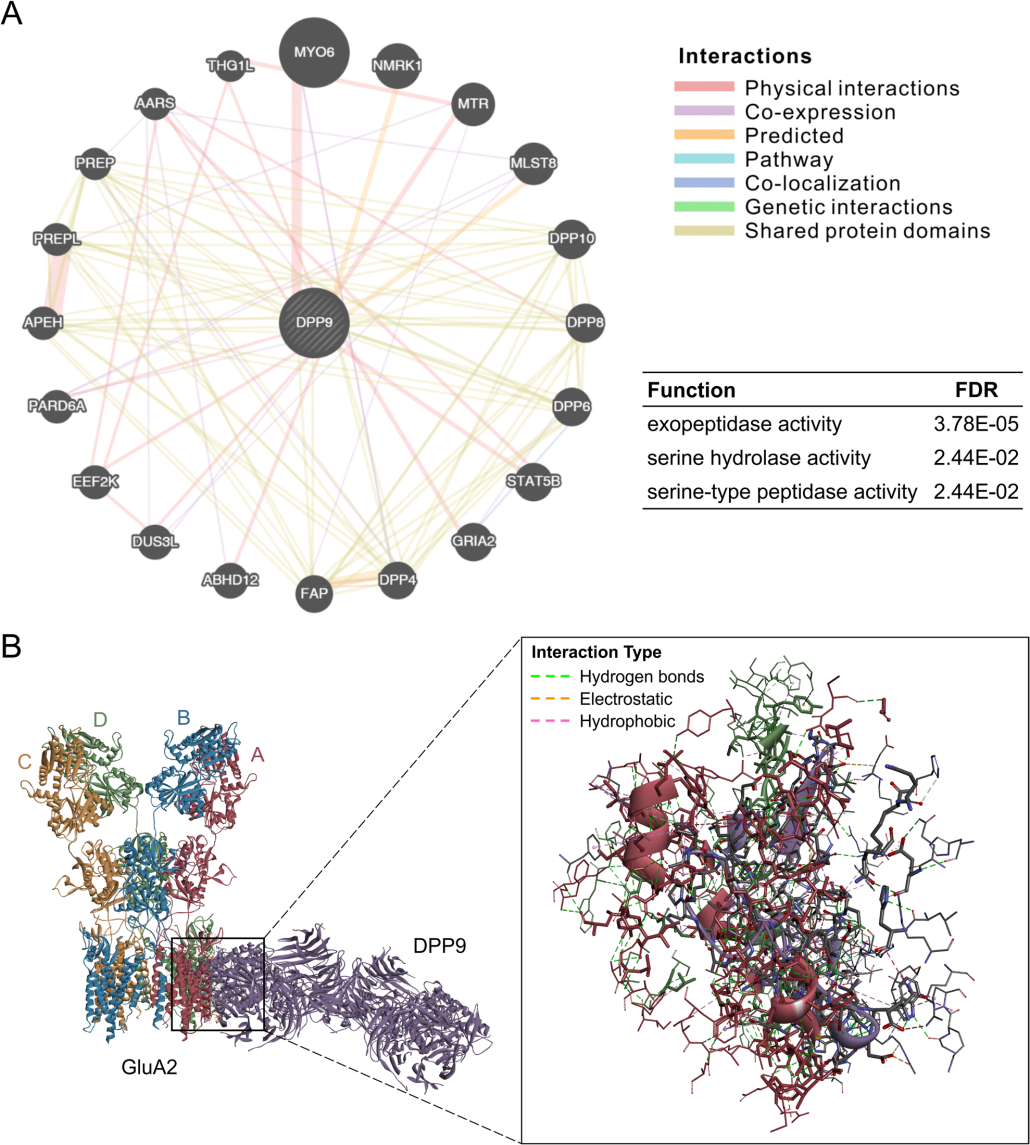
GeneMANIA function analysis of DPP9 and a possible GluA2-DPP9 docking simulation. **A** Interaction network illustrating physical interactions, co-expression, predicted interactions, pathways, co-localization, genetic interactions, and shared protein domains of DPP9. **B** A top-ranked model from the GluA2-DPP9 docking simulation.

To investigate the potential role of DPP9 in the central nervous system, we used the GeneMANIA online tool to conduct a functional analysis of DPP9. The findings revealed physical interactions between DPP9 and key proteins such as MYO6, MTR, GRIA2 (also recognized as GluA2), and STAT5B, which are closely associated with learning and memory processes [67–71] (Fig. 4A). Notably, GluA2 is a well-documented gene implicated in memory expression and maintenance [30, 72, 73]. Moreover, a comprehensive human interactome has corroborated the interaction between DPP9 and GluA2 [74]. To further analyze this interaction, we assessed the molecular docking of GluA2 (PDB ID: 6DM1) with DPP9 (PDB ID: 6EOQ) in humans. Considering the substantial molecular weight of GluA2, we excluded its extracellular domain (the ATD) and utilized the TMD or LBD in combination with the TMD (LBD + TMD) for separate docking with DPP9. A previous study revealed that removal of the ATD of GluA1 does not affect membrane topology or surface delivery [75]. Given the high homology of AMPARs, we hypothesized that removal of the ATD of GluA2 would also not affect its primary structure. In addition, removal of the ATD of GluN2 does not eliminate its pH sensitivity or alter its active site for binding to downstream proteins [76]. Therefore, we believe that this removal does not influence the structure of GluA2 or change its active binding site. Molecular docking was executed using Discovery Studio, ZDOCK Server, LZerD Server, and GRAMM. Notably, only GRAMM yielded significant results. The docking results for the TMD or LBD + TMD of GluA2 with DPP9 are presented in Fig. S4. After ruling out models showing an implausible transmembrane orientation of DPP9, the highest-ranking docking model was Model 2 of the GluA2 TMD with DPP9, with the specific binding interactions highlighted in the enlarged illustration (Fig. 4B).

### Inhibition of DPP9 Disturbs Contextual Fear Memory Retrieval at 48 h but not at 9 days

**Fig. 5:**
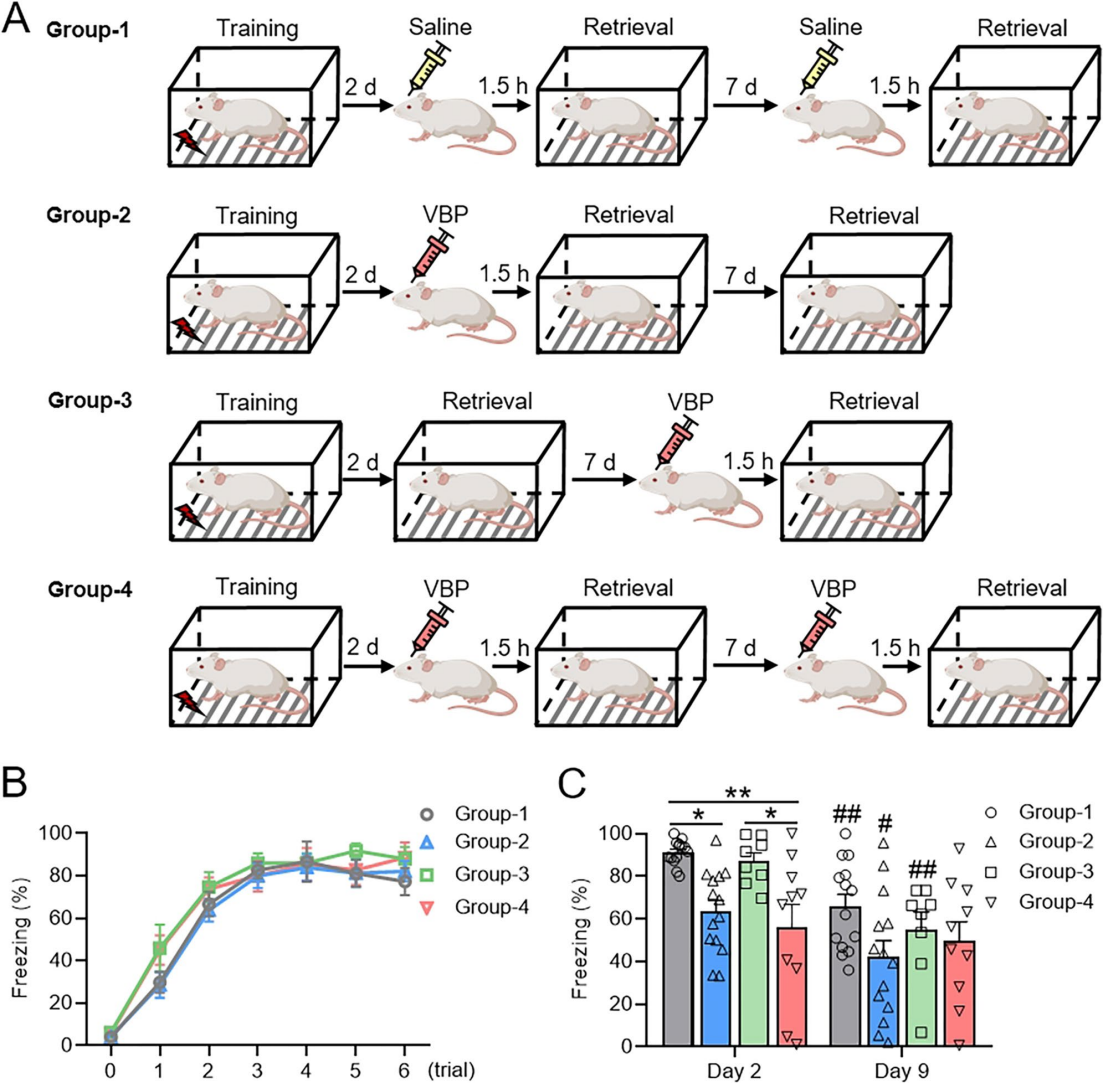
Pharmacological inhibition of DPP9 significantly disturbs memory retrieval at 2 days after learning but not at 9 days. **A** Diagram illustrating three types of VBP administration groups and a saline administration control group. **B** Learning curves of the four groups. **C** The memory retrieval performance of rats at 2 days after learning. **D** The memory retrieval performance of rats at 9 days after learning. For group 1, *n =* 14; group 2, *n =* 14; group 3, *n =* 8; group 4, *n =* 10. For learning curve statistical comparisons, two-way RM ANOVA was followed by Bonferroni’s post-tests. For memory retrieval statistical comparisons, one-way ANOVA was followed by Bonferroni’s post-tests. *P < 0.05, **P < 0.01, ***P < 0.005. Values are expressed as the mean ± SEM.

The physical interaction between GluA2 and DPP9 highlights the functional significance of these proteins. Given that GluA2-dependent AMPAR trafficking constitutes a fundamental cellular mechanism for memory retrieval and reconsolidation, we speculated that DPP9 might play a role in these processes. To further investigate the involvement of DPP9 in memory retrieval and retrieval-induced reconsolidation, we designed four pharmacological administration groups. The control group received saline at both 48 h and 9 days during the retrieval period. In the 48-h administration group, VBP was administered only at 48 h but not at 9 days. Conversely, in the 9-day administration group, VBP was not administered at 48 h but was only administered at 9 days. The 48-h and 9-day administration groups received VBP at both 48 h and 9 days during retrieval (Fig. 5A). All four groups of rats underwent contextual fear conditioning, and their performance did not significantly differ (Fig. 5B). Upon retrieval, VBP significantly disrupted fear memory retrieval when VBP was administered on day 2 in the comparisons between group 1 and group 2 or group 4 on day 2 (Fig. 5C). In contrast, there was no significant effect of VBP on memory retrieval when it was administered on day 9 in the comparison between group 1 and group 3 on day 9 (Fig. 5C). These pharmacological results strongly suggest that DPP9 is intricately involved in memory retrieval on day 2 but not on day 9. Notably, there was a significant decrease in the memory retrieval level on day 9 compared with that on day 2 in the control group. Fig. S5 shows that the memory retrieval level was equivalent on day 2 and day 9, suggesting that the above decrease in the control group was due to the repeated retrieval of memory. Furthermore, our results indicated that, regardless of the administration of VBP on day 2, retrieval attenuated memory, as indicated in groups 1–3 (Fig. 5C). Notably, in group 4, when VBP was administered on day 2 and day 9, memory attenuation on day 9 retrieval caused by the day 2 retrieval was obstructed (Fig. 5C); however, in groups 2 and 3, memory retrieval on day 9 was lower than that on day 2 when VBP was administered once on day 2 or day 9. The above findings suggest that administrations of VBP twice on day 2 and day 9 may affect memory reconsolidation, resulting in the disappearance of memory decay caused by repeated retrieval. However, the experimental design used here was not sufficient to determine whether DPP9 directly participates in the reconsolidation process. It is necessary to intervene in DPP9 only in the reconsolidation stage after retrieval to draw a definite conclusion. Additional information, such as the body weight of each group and Nissl staining for cannula location identification, are provided in Fig. S6.

### DPP9 Overexpression Resists Retrieval-Induced Fear Memory Attenuation

**Fig. 6:**
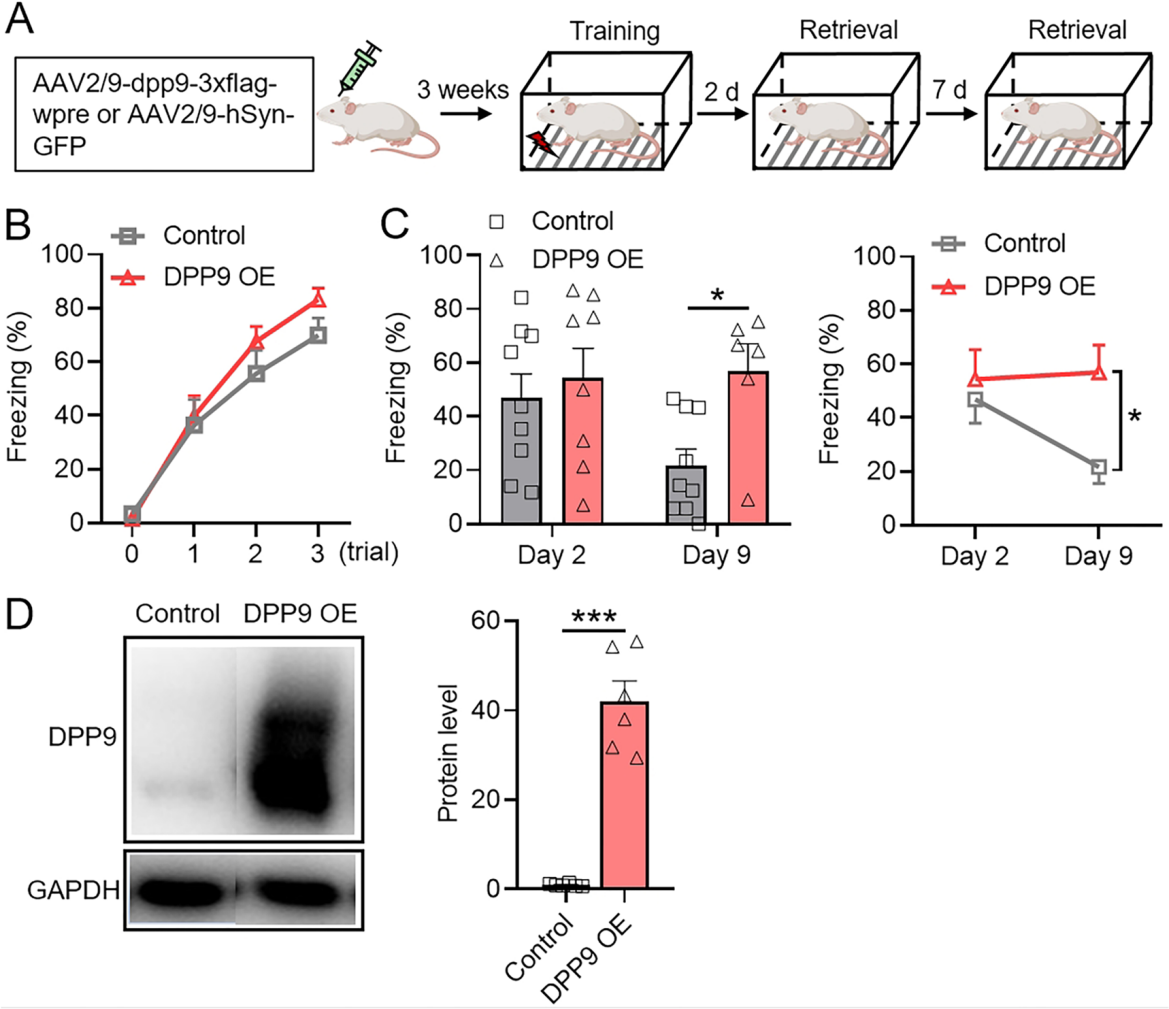
DPP9 overexpression counters retrieval-induced fear memory attenuation. **A** Virus injection and behavioral scheme. **B** Learning curves of the DPP9 over-expression group (*n =* 9) and the control group (*n =* 9). **C** The memory retrieval performance of rats at 2 and 9 days after learning (*n =* 9 for the control group both on 2- and 9-day retrieval, *n =* 8 for the DPP9 overexpression group on 2-day retrieval, *n =* 6 for DPP9 overexpression group on 9-day retrieval); two-way RM ANOVA followed by Bonferroni’s post-tests; *P < 0.05. **D** DPP9 protein expression level of the overexpression group (*n =* 6) and control group (*n =* 6); unpaired *t*-test; ***P < 0.001. Values are expressed as the mean ± SEM.

Building upon the above findings, we sought to investigate whether overexpression of the DPP9 protein could enhance the retrieval performance of retrieval-induced reconsolidation on day 2. Given that the learning and retrieval performance in the previous behavior protocol exhibited a ceiling effect, we opted to modify the protocol to a milder version (0.55 mA for 3 trials of foot shocks). The rats received injections of the DPP9-overexpressing virus into the bilateral hippocampus, and after three weeks of viral expression, they underwent contextual fear conditioning. Fear memory retrieval was assessed at 2 and 9 days after learning (Fig. 6A). Both the DPP9 overexpression group and the control group exhibited proficient memory learning performance (Fig. 6B). Although there was no significant difference in the learning curves between the two groups, it cannot be ruled out that DPP9 has no effect on the learning process, as the DPP9 overexpression group seemed to have better performance than the control group. There was no significant difference in performance between the DPP9 overexpression group and the control group at 2 days of memory retrieval. However, at the 9-day retrieval point, the DPP9 overexpression group had a significantly greater retrieval level than the control group (Fig. 6C, left). The memory trend of the DPP9 overexpression group was notably greater than that of the control group (Fig. 6C, right). These results indicated that DPP9 overexpression does not increase the fear memory retrieval level at 48 h but mitigates its decline after retrieval, suggesting that the overexpression of DPP9 may impact the reconsolidation of fear memory at 48 h and, consequently, resist the attenuation of fear memory induced by retrieval [77, 78]. The western blot results further confirmed a significant increase in the DPP9 expression level in the GFP-positive tissue of the DPP9 overexpression group over that in the control group (Figs 6D, Fig. S7).

## Discussion

The formation and consolidation of memory are well-established as dynamic, phase-dependent regulatory processes. In this study, we addressed these issues through systematic analysis of temporal transcription profiles of fear memory retrieval at 0.5, 24, 48, and 72 hours after learning. The retrieval at different time points revealed substantial DEGs, which were significantly enriched in different pathways at various time points. STEM analysis revealed three significantly enriched clusters with distinct waves of expression. Further screening of the most significant cluster of 49 characteristic DEGs identified nine genes, most of which have established associations with learning and memory. Combined with the results of the behavioral qPCR verification, we propose that the expression of this cluster 49 DEGs may play a specific role induced by 48-h fear memory retrieval rather than the formation and consolidation of memory, at least under the experimental conditions of our study. These results substantiate that the transcriptional regulation induced by recent memory retrieval is a time-dependent and highly dynamic process. We subsequently selected *DPP9*, a gene not previously reported in the field of learning and memory, for further functional analysis and animal experiments. Protein docking simulations suggested a potential interaction between DPP9 and GluA2. Pharmacological intervention and viral vector-mediated overexpression of DPP9 confirmed its role in memory retrieval or reconsolidation.

We identified three clusters of transcripts exhibiting temporally dynamic characteristics and confirmed the involvement of screened genes specifically induced by 48-h fear memory retrieval. These findings strongly suggest the existence of time-dependent features of dynamic regulation induced by recent memory retrieval. In our study, retrieval at 48 h was associated with the greatest number of DEGs. Consequently, we focused on cluster 49, characterized by continuously upregulated gene expression from 0.5 h to 48 h, followed by modestly downregulated gene expression from 48 h to 72 h, for further investigation. Screening DEGs that followed the expression trend of cluster 49 revealed that most of these genes have been previously reported to be involved in learning and memory. These findings suggest that 48-h memory retrieval may necessitate the coexpression of specific genes associated with learning and memory. Further experiments are warranted to ascertain whether these genes act in concert with specific signaling pathways. While the other two significantly enriched clusters are noteworthy, our focus remained on cluster 49. We selected the characteristic DEGs of cluster 49, and the genes significantly upregulated at the 48-h time point in the KEGG pathway for qPCR validation. The qPCR results suggested that these genes function as phase-dependent retrieval-induced genes, as their expression levels were significantly elevated when both training and retrieval conditions were met and at the 48-h time point. However, we cannot definitively exclude the possibility that these genes play a role at other time points or in other memory processes. Rigorous experiments are essential to confirm or refute these possibilities. As controls in the qPCR experiment, we selected genes that did not meet the above-selected conditions (Fig. 3F–H). Notably, *Zif268* expression was elevated in both the RET group and the No-RET group at 48 h, with particularly high expression in the No-RET group at multiple time points. In addition, *Npase* and *Fos* also displayed a certain degree of elevated expression at 48 h. These findings suggest that phase-specific transcription factors, such as Zif268, may act as rapid-response genes, initiating multiple waves of transcription that trigger signal cascades and ultimately contribute to the molecular preparations for memory reconsolidation, as found with DPP9 in this study [25, 79–82]. However, further experiments are warranted to validate this hypothesis. Notably, *Gapdh* expression seems to decrease with time; thus, *Gapdh* may not be the most suitable reference gene in this study, and more reference genes should be selected as controls for further verification.

Our study has made significant contributions to our understanding of memory retrieval-induced molecular events, shedding light on the role of DPP9 in reconsolidation. The widespread expression of DPP9 in the brain [64–66], coupled with its association with molecules such as neuropeptide Y and calreticulin, is implicated in learning and memory, suggesting the importance of DPP9 in memory processes [83–85]. Notably, the documented interaction between DPP9 and GluA2 reported previously [74], coupled with our docking results, suggests the potential involvement of DPP9 in GluA2-AMPAR signaling processes. The dynamic characteristics of AMPARs align well with the rapid temporal scale features of memory retrieval [59]. The heterogeneous composition, kinetics, and number of AMPARs accompanying AMPAR trafficking may underlie the phase-dependent synaptic plasticity mechanism of memory reconsolidation [31, 86–89]. The regulation of dynamic AMPAR trafficking in various synaptic plasticity processes involves interactions with different proteins, such as GRIP, PICK1, and NSF, which bind to the intracellular carboxy-tail of GluA2 [90–93]. Notably, DPP9, a soluble intracellular protein, has been reported to be translocated to the surface of immune cells under certain conditions [94]. Our GluA2-DPP9 docking simulation results predict the bonding of DPP9 with the C-terminus of GluA2. In light of the function of DPP9 in memory retrieval and reconsolidation found in our study, we hypothesize that DPP9 may act as a candidate that interacts with GluA2 to participate in the regulation of AMPAR trafficking. The inhibition of DPP9 may cause the endocytosis of GluA2-containing Ca^2+^-impermeable AMPARs, underlying the mechanism of poor retrieval performance in our study; the overexpression of DPP9 may cause the exchange of GluA2-lacking CP-AMPARs to GluA2-containing CI-AMPARs at the synapse by the interaction of the GluA2 subunit with DPP9, underlying the mechanism of enhanced memory reconsolidation shown in our results. Although there is no experimental evidence here, this is still an intriguing direction that warrants further experimental testing. Notably, we cannot rule out the possibility that DPP9 may be located in the cell body and regulate learning and memory processes through other mechanisms. We found that the impact of VBP on fear memory retrieval differed between day 2 and day 9. On the one hand, this may be due to a decrease in the transcription level of dpp9, so the involvement of DPP9 in this process decreases from day 2 to day 9. On the other hand, this discrepancy may be attributed to system consolidation, as maturation of memory engrams occurs from the hippocampus to more brain regions, such as the prefrontal cortex [95]. Therefore, the effect of hippocampal DPP9 intervention on day 9 may be weaker than that on day 2. However, our study did not investigate DPP9 in other brain regions, such as the prefrontal cortex and amygdala. Exploring the effects of DPP9 intervention in multiple brain regions at different time points will help us gain a deeper understanding of the results in our study and help us understand how DPP9 dynamically participates in the process of memory consolidation and reconsolidation.

However, several unresolved issues remain. First, while we identified time-dependent dynamic gene waves during memory retrieval, the regulatory mechanisms of these genes by transcription factors with temporal evolution and their participation in the memory retrieval process remain unclear. Second, there is room for improvement regarding DPP9 intervention. The VBP used in our study is a nonspecific inhibitor of DPP9 [96], and currently, there is no specific inhibitor available [97, 98]. Moreover, the lack of temporal specificity in the intervention of DPP9 during the retrieval phase calls for the development of a spatiotemporally specific genetic manipulation system, allowing for the precise knockdown or overexpression of DPP9 in a defined time window to determine its exact role in a specific memory phase. Finally, we sought to elucidate the signaling pathways associated with memory retrieval and reconsolidation in which DPP9 may play a role. Although our docking results suggest AMPAR trafficking regulation as a potential mechanism, the absence of experimental evidence necessitates further investigation. Moreover, exploring the enzymatic activity of DPP9 on neuromodulators within the central nervous system offers an intriguing avenue for future research.

In summary, our transcriptome results revealed characteristic molecular events induced by fear memory retrieval at different time points, indicating the temporally dynamic regulation of the memory retrieval-induced process. Among these events, we identified the function of DPP9 in fear memory retrieval and reconsolidation. Further exploration of the molecular signaling and synaptic plasticity mechanisms of DPP9 in memory retrieval will enhance our understanding of the temporal transcriptional regulation of retrieval-induced memory reprocessing. Our findings suggest that DPP9 has great potential as a new molecular target for the treatment of memory disturbance disorders.

## Supplementary Information

Below is the link to the electronic supplementary material.

Supplementary file1 (PDF 1885 KB)
