# Supplementary material for: Time-Dependent Transcriptional Dynamics of Contextual Fear Memory Retrieval Reveals the Function of Dipeptidyl Peptidase 9 in Reconsolidation
Source: Neurosci Bull. 2024 Dec 2;41(1):16–32. doi: 10.1007/s12264-024-01324-w (PMC11748732; doi:10.1007/s12264-024-01324-w)
Supplement: Supplementary file 1 — Supplementary file1 (PDF 1885 KB) [file 12264_2024_1324_MOESM1_ESM.pdf]

Supplementary Materials

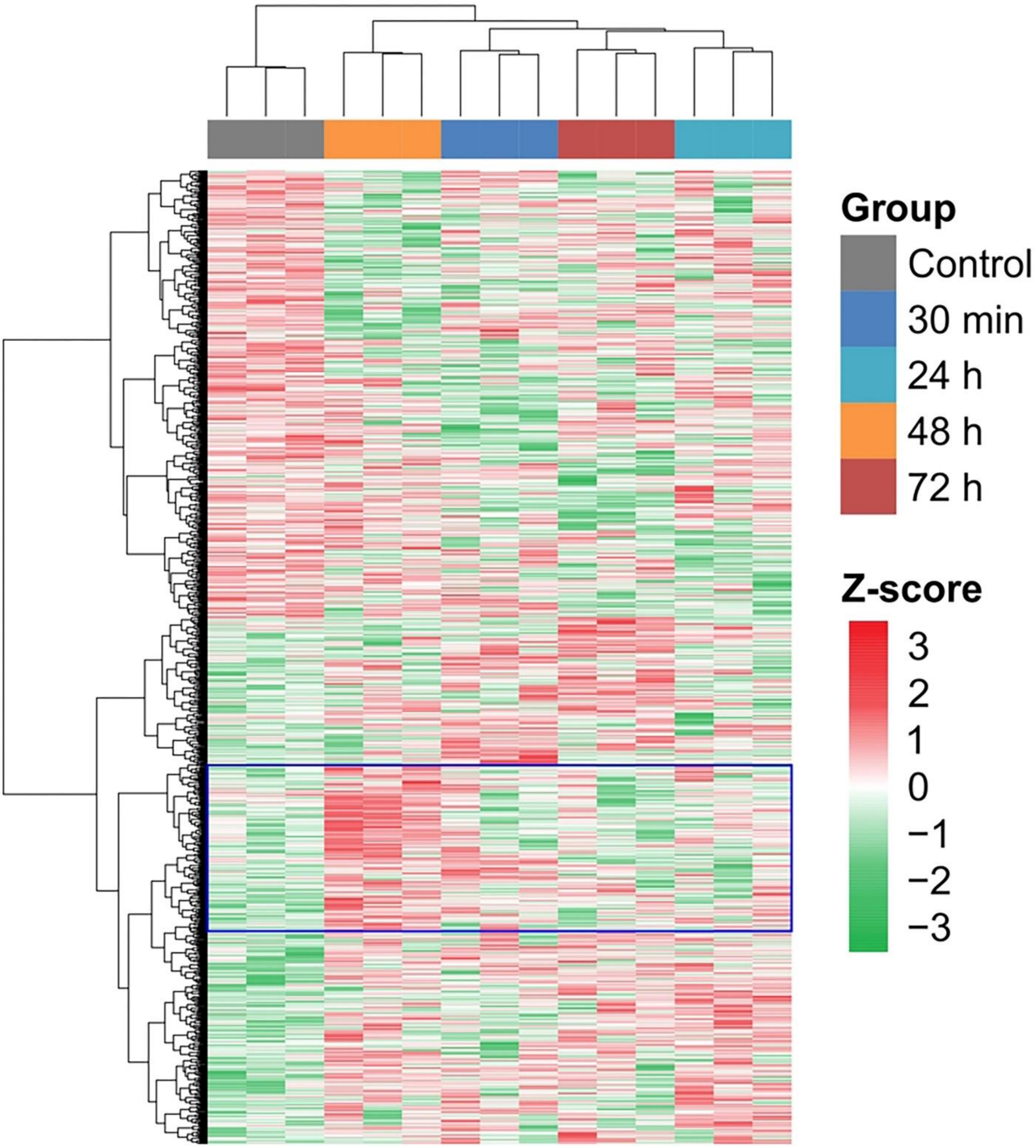

Fig. S1. Expression profiles of the DEGs in four comparisons (n = 1217) in all samples. All gene expression values are converted to z-scores. The blue boxes indicate that a considerable number of genes show higher expression in the 48-h retrieval group than all other groups.

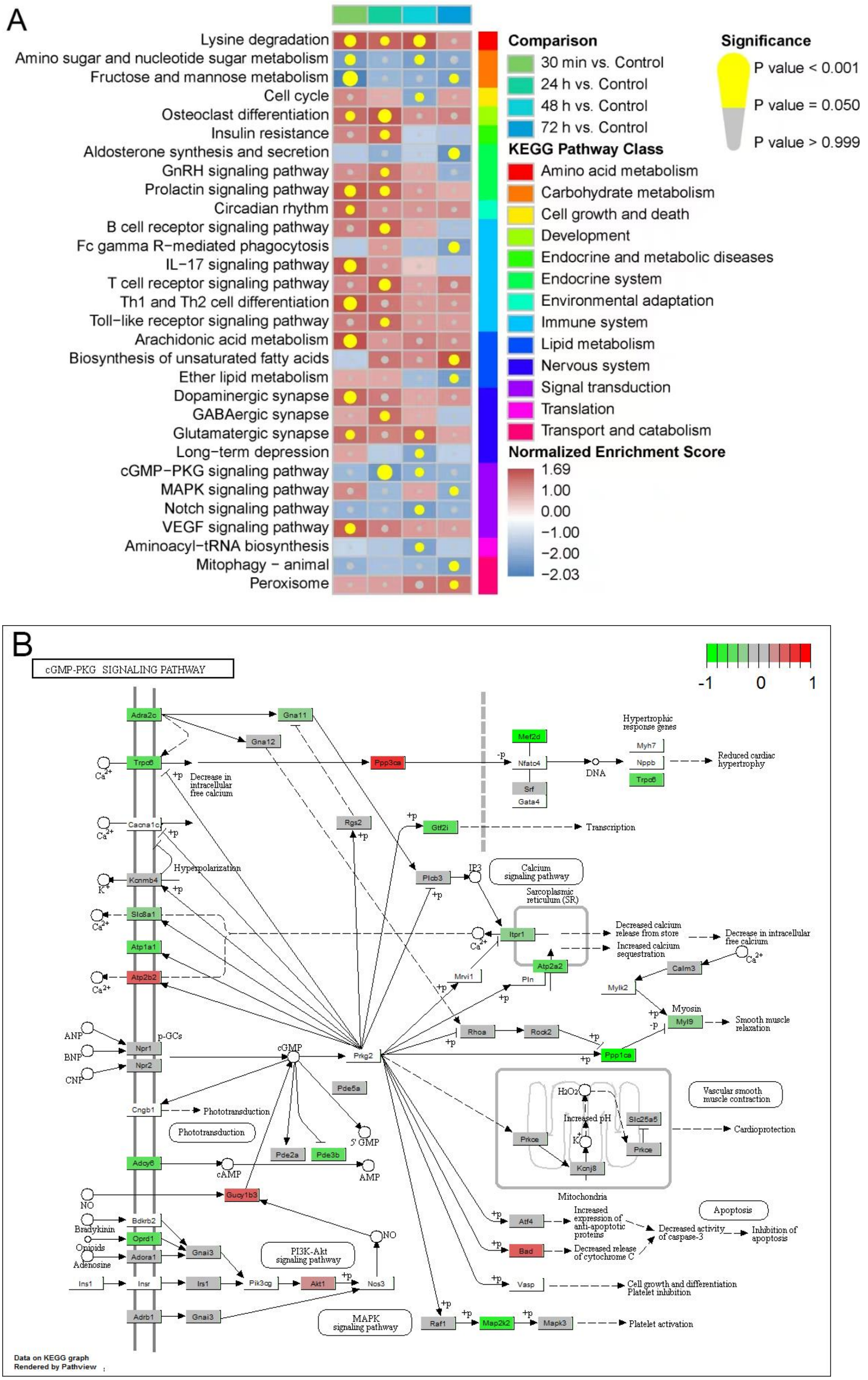
A
Lysine degradation
Amino sugar and nucleotide sugar metabolism
Fructose and mannose metabolism
Cell cycle
Osteoclast differentiation
Insulin resistance
Aldosterone synthesis and secretion
GnRH signaling pathway
Prolactin signaling pathway
Circadian rhythm
B cell receptor signaling pathway
Fc gamma R−mediated phagocytosis
IL−17 signaling pathway
T cell receptor signaling pathway
Th1 and Th2 cell differentiation
Toll−like receptor signaling pathway
Arachidonic acid metabolism
Biosynthesis of unsaturated fatty acids
Ether lipid metabolism
Dopaminergic synapse
GABAergic synapse
Glutamatergic synapse
Long−term depression
cGMP−PKG signaling pathway
MAPK signaling pathway
Notch signaling pathway
VEGF signaling pathway
Aminoacyl−tRNA biosynthesis
Mitophagy − animal
Peroxisome
Comparison
30 min vs. Control
24 h vs. Control
48 h vs. Control
72 h vs. Control
KEGG Pathway Class
Amino acid metabolism
Carbohydrate metabolism
Cell growth and death
Development
Endocrine and metabolic diseases
Endocrine system
Environmental adaptation
Immune system
Lipid metabolism
Nervous system
Signal transduction
Translation
Transport and catabolism
Normalized Enrichment Score
1.69
1.00
0.00
−1.00
−2.00
−2.03
Significance
P value < 0.001
P value = 0.050
P value > 0.999
B
cGMP-PKG SIGNALING PATHWAY
-1
0
1
Adra2c
Gna11
Gna12
Trpc6
Ppp3ca
Mef2d
Nfatc4
Srf
Gata4
DNA
Hypertrophic response genes
Myh7
Nppb
Trpc6
Reduced cardiac hypertrophy
Decrease in intracellular free calcium
Cacna1c
Rgs2
Gtf2i
Transcription
Hyperpolarization
Kcnmb4
Plcb3
IP3
Calcium signaling pathway
Sarcoplasmic reticulum (SR)
Slc8a1
Itpr1
Decreased calcium release from store
Increased calcium sequestration
Decrease in intracellular free calcium
Atp1a1
Atp2a2
Calm3
Atp2b2
Mrvi1
Pln
Mylk2
Myosin
Myl9
Smooth muscle relaxation
ANP
BNP
CNP
p-GCs
Npr1
Npr2
cGMP
Prkg2
Rhoa
Rock2
Ppp1ca
Pde5a
Cngb1
Phototransduction
Vascular smooth muscle contraction
Prkce
Slc25a5
Increased pH
Kcnj8
Cardioprotection
Mitochondria
Pde2a
Pde3b
5' GMP
Adcy6
cAMP
AMP
NO
Gucy1b3
Apoptosis
Bradykinin
Bdkrb2
Opioids
Oprd1
Adenosine
Adora1
Gnai3
PI3K-Akt signaling pathway
Atf4
Increased expression of anti-apoptotic proteins
Decreased activity of caspase-3
Inhibition of apoptosis
Bad
Decreased release of cytochrome C
Ins1
Insr
Irs1
Pik3cg
Akt1
Nos3
Vasp
Cell growth and differentiation
Platelet inhibition
Adrb1
MAPK signaling pathway
Raf1
Map2k2
Mapk3
Platelet activation
Data on KEGG graph
Rendered by Pathview

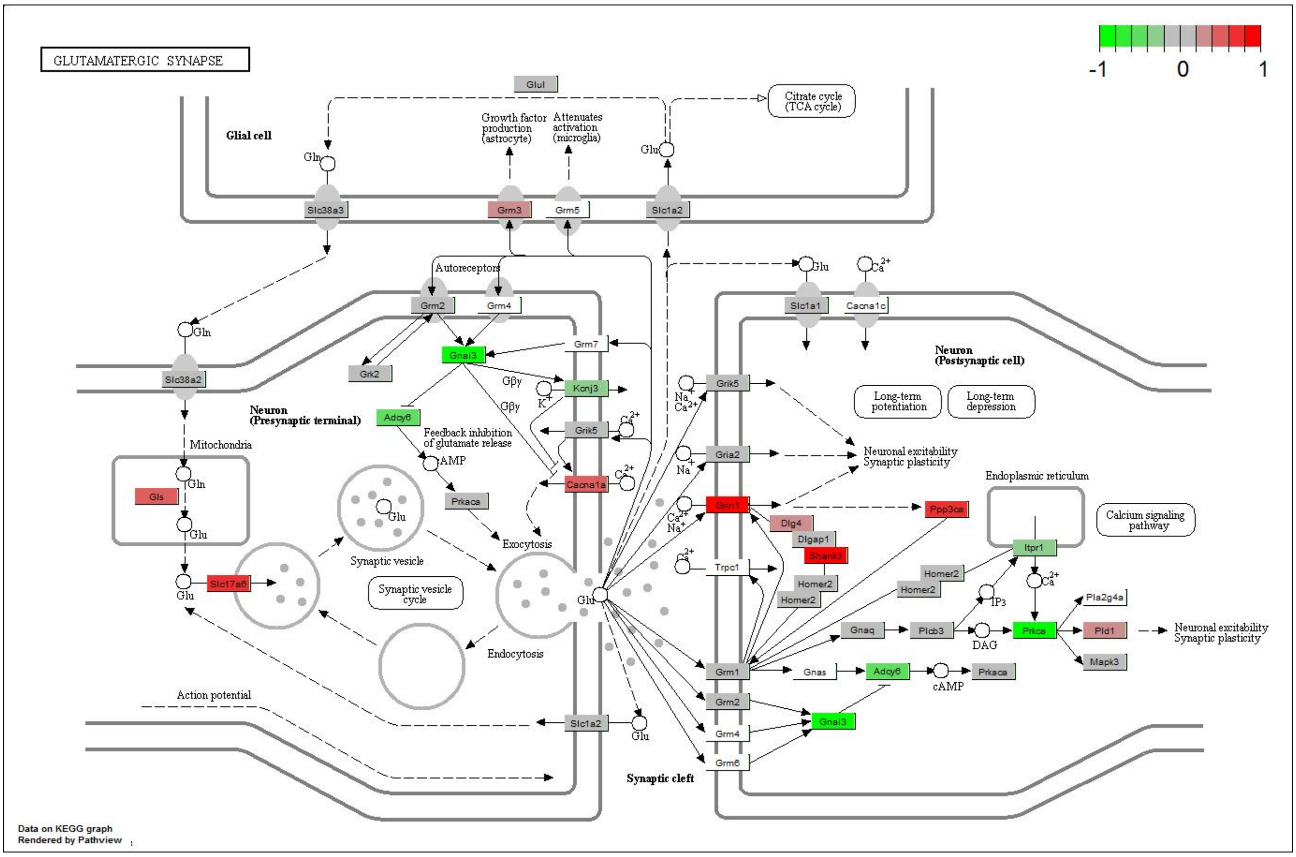

GLUTAMATERGIC SYNAPSE
-1
0
1
Glul
Citrate cycle (TCA cycle)
Glial cell
Growth factor production (astrocyte)
Attenuates activation (microglia)
Gln
Glu
Slc38a3
Grm3
Grm5
Slc1a2
Autoreceptors
Grm2
Grm4
Glu
Ca2+
Slc1a1
Cacna1c
Gln
Grm7
Gnai3
Grk2
Slc38a2
Gβγ
Kcnj3
K+
Neuron (Postsynaptic cell)
Neuron (Presynaptic terminal)
Adcy6
Gβγ
Grik5
Ca2+
Grik5
Na+
Ca2+
Long-term potentiation
Long-term depression
Mitochondria
Feedback inhibition of glutamate release
AMP
Cacna1a
Gria2
Na+
Neuronal excitability
Synaptic plasticity
Gln
Gls
Glu
Prkaca
Endoplasmic reticulum
Synaptic vesicle
Exocytosis
Grin1
Ppp3ca
Calcium signaling pathway
Dlg4
Dlgap1
Shank3
Itpr1
Trpc1
Homer2
Homer2
Homer2
Homer2
IP3
Ca2+
Slc17a6
Synaptic vesicle cycle
Glu
Gnaq
Plcb3
DAG
Prkca
Pla2g4a
Pld1
Neuronal excitability
Synaptic plasticity
Mapk3
Endocytosis
Grm1
Gnas
Adcy6
cAMP
Prkaca
Grm2
Gnai3
Action potential
Slc1a2
Glu
Grm4
Grm6
Synaptic cleft
Data on KEGG graph
Rendered by Pathview

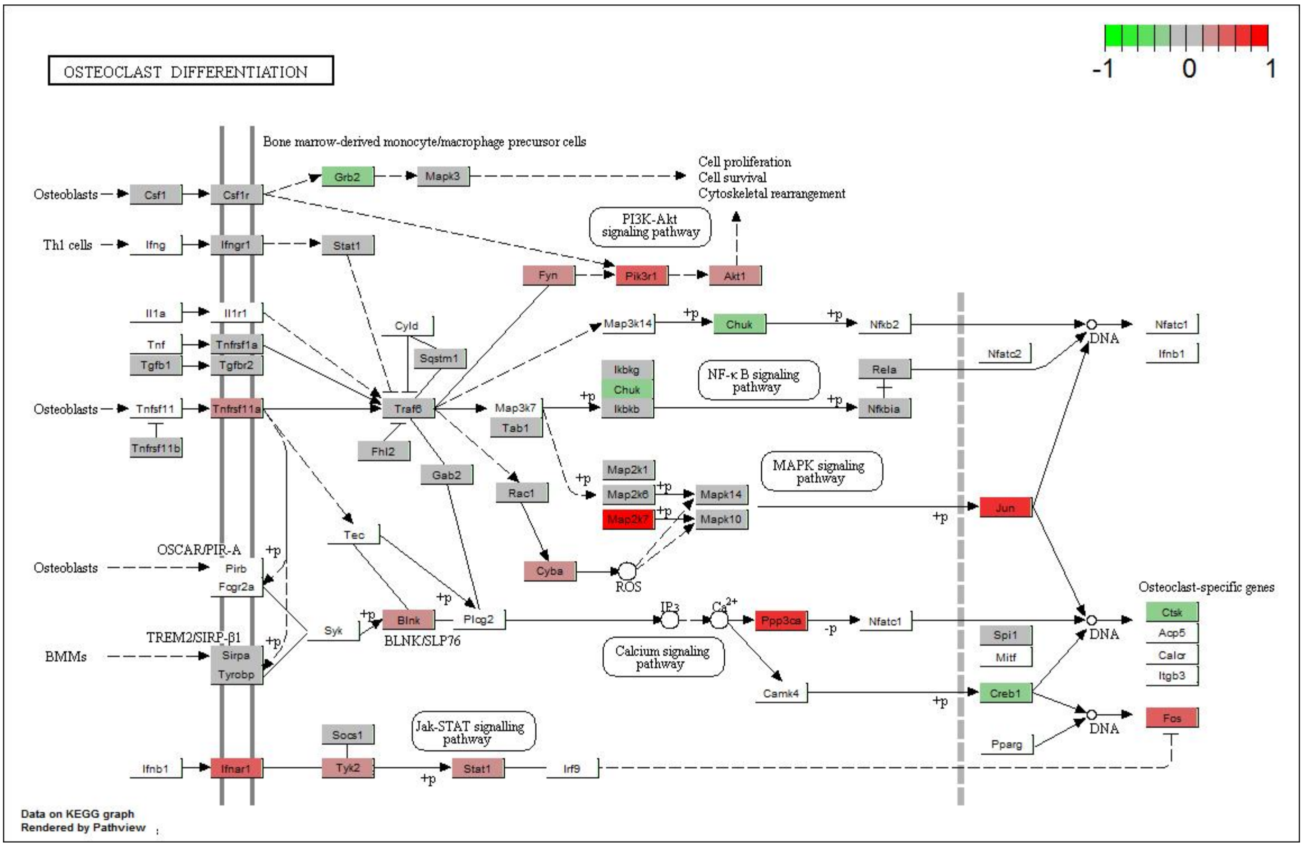

OSTEOCLAST DIFFERENTIATION
-1
0
1
Bone marrow-derived monocyte/macrophage precursor cells
Grb2
Mapk3
Cell proliferation
Cell survival
Cytoskeletal rearrangement
Osteoblasts
Csf1
Csf1r
PI3K-Akt signaling pathway
Th1 cells
Ifng
Ifngr1
Stat1
Fyn
Pik3r1
Akt1
Il1a
Il1r1
Cyld
Map3k14
+p
Chuk
+p
Nfkb2
DNA
Nfatc1
Tnf
Tnfrsf1a
Sqstm1
Nfatc2
Ifnb1
Tgfb1
Tgfbr2
Ikbkg
Chuk
NF-κB signaling pathway
Rela
Osteoblasts
Tnfsf11
Tnfrsf11a
Traf6
Map3k7
+p
Ikbkb
+p
Nfkbia
Tnfrsf11b
Tab1
Fhl2
Gab2
Map2k1
MAPK signaling pathway
Rac1
+p
Map2k6
+p
Mapk14
Map2k7
+p
Mapk10
Jun
+p
Tec
OSCAR/PIR-A
+p
Osteoblasts
Pirb
Cyba
ROS
Osteoclast-specific genes
Fcgr2a
+p
Ctsk
+p
Blnk
Plcg2
IP3
Ca2+
Ppp3ca
Nfatc1
DNA
Syk
-p
TREM2/SIRP-β1
BLNK/SLP76
Spi1
Acp5
BMMs
+p
Sirpa
Calcium signaling pathway
Mitf
Calcr
Tyrobp
Itgb3
Camk4
Creb1
+p
Socs1
Jak-STAT signalling pathway
DNA
Fos
Pparg
Ifnb1
Ifnar1
Tyk2
Stat1
Irf9
+p
Data on KEGG graph
Rendered by Pathview

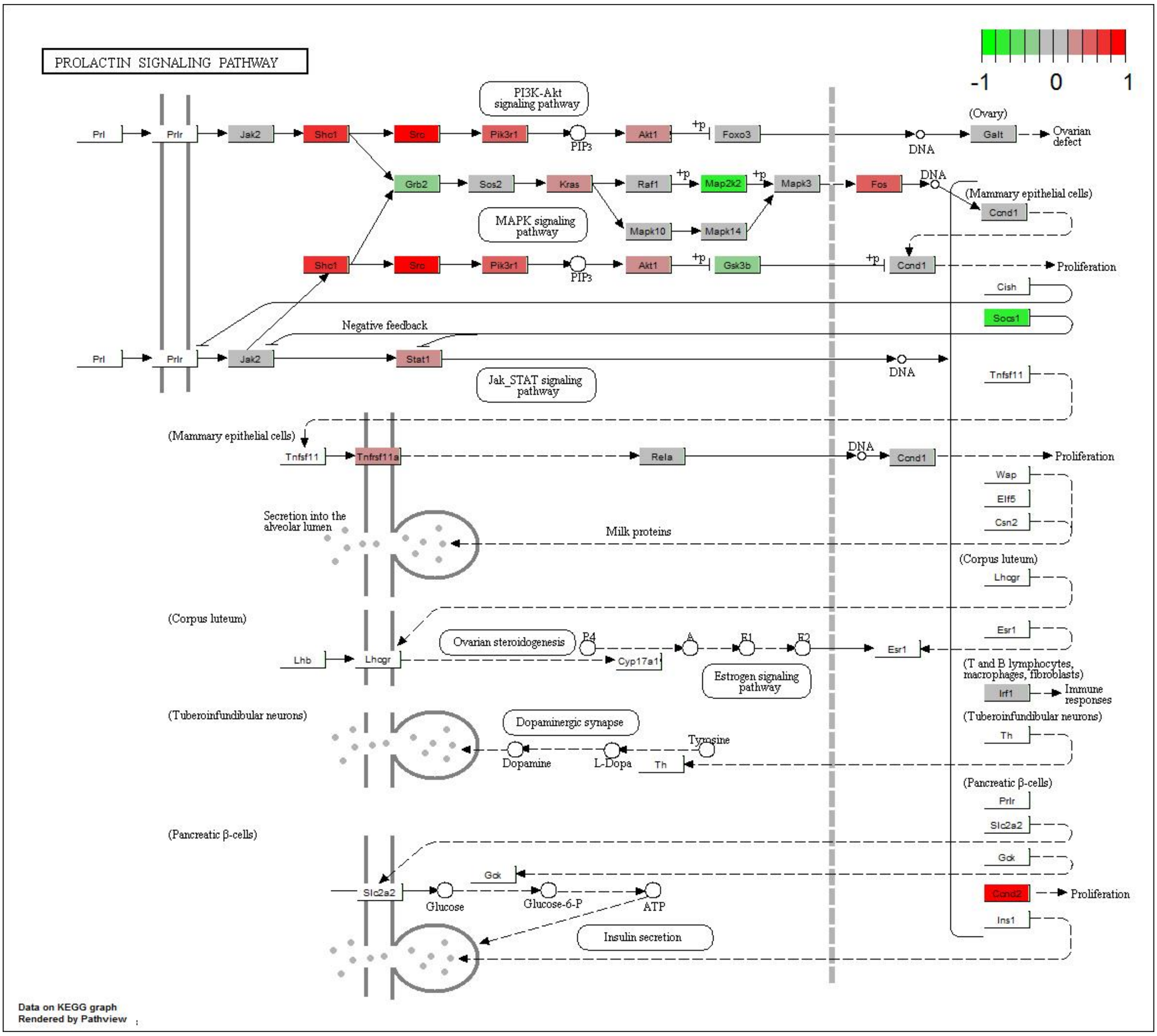

Fig. S2. Gene set enrichment analysis of post-learning retrieval groups at different times. **A** KEGG pathway analysis of post-learning retrieval groups at different times compared with the control group. Red boxes, upregulated pathways; blue boxes, downregulated pathways; yellow circles, significantly enriched pathways. **B** Visualization of the glutamatergic synapse, cGMP-PKG signaling pathway, prolactin signaling pathway, and osteoclast differentiation pathway for 48-h retrieval *vs* control. Red boxes, upregulated genes; blue boxes, downregulated genes; gray boxes unchanged genes.

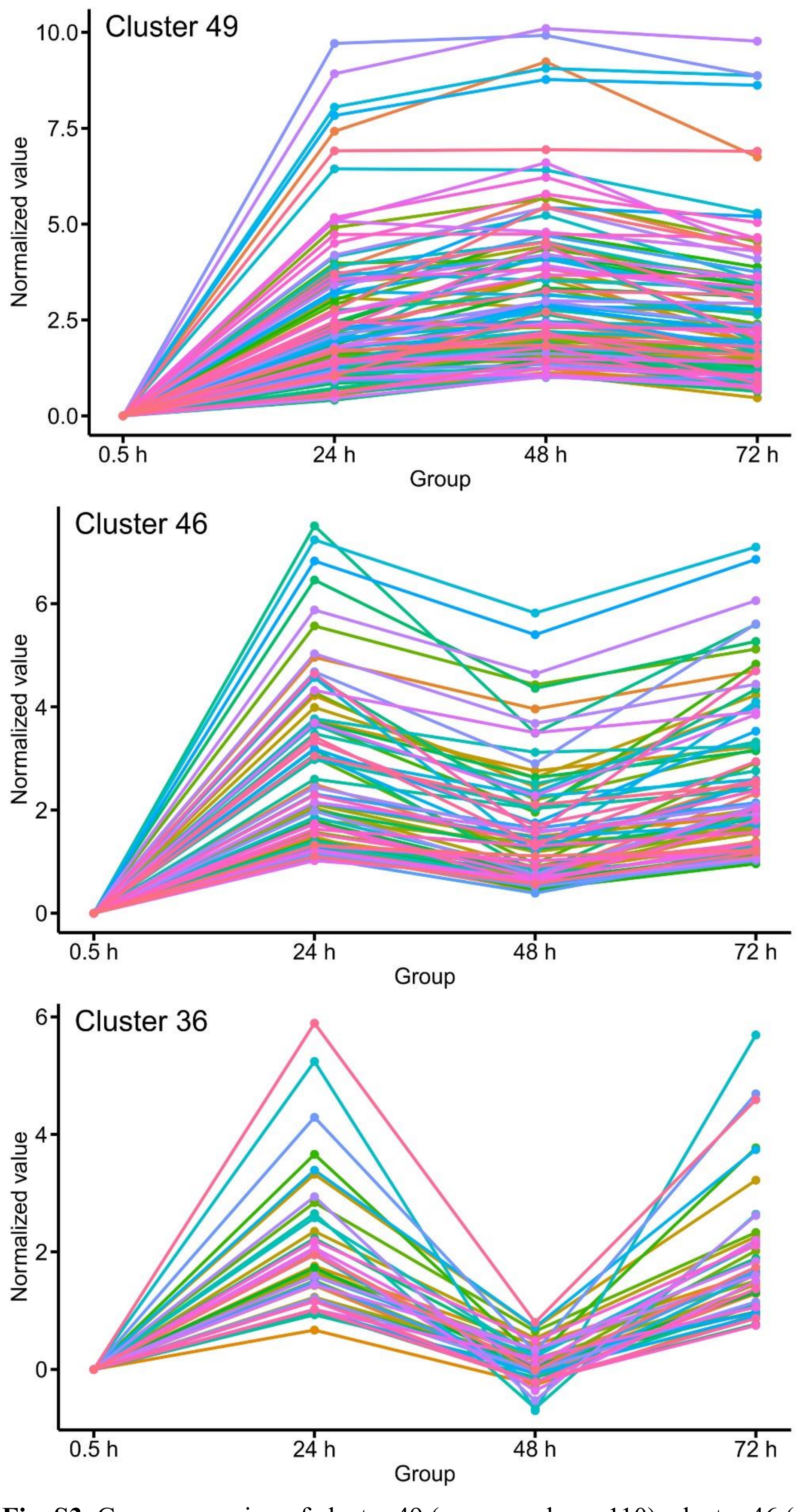

Fig. S3. Gene expression of cluster 49 (gene numbers: 110), cluster 46 (gene numbers: 74), and cluster 36 (gene numbers: 43).

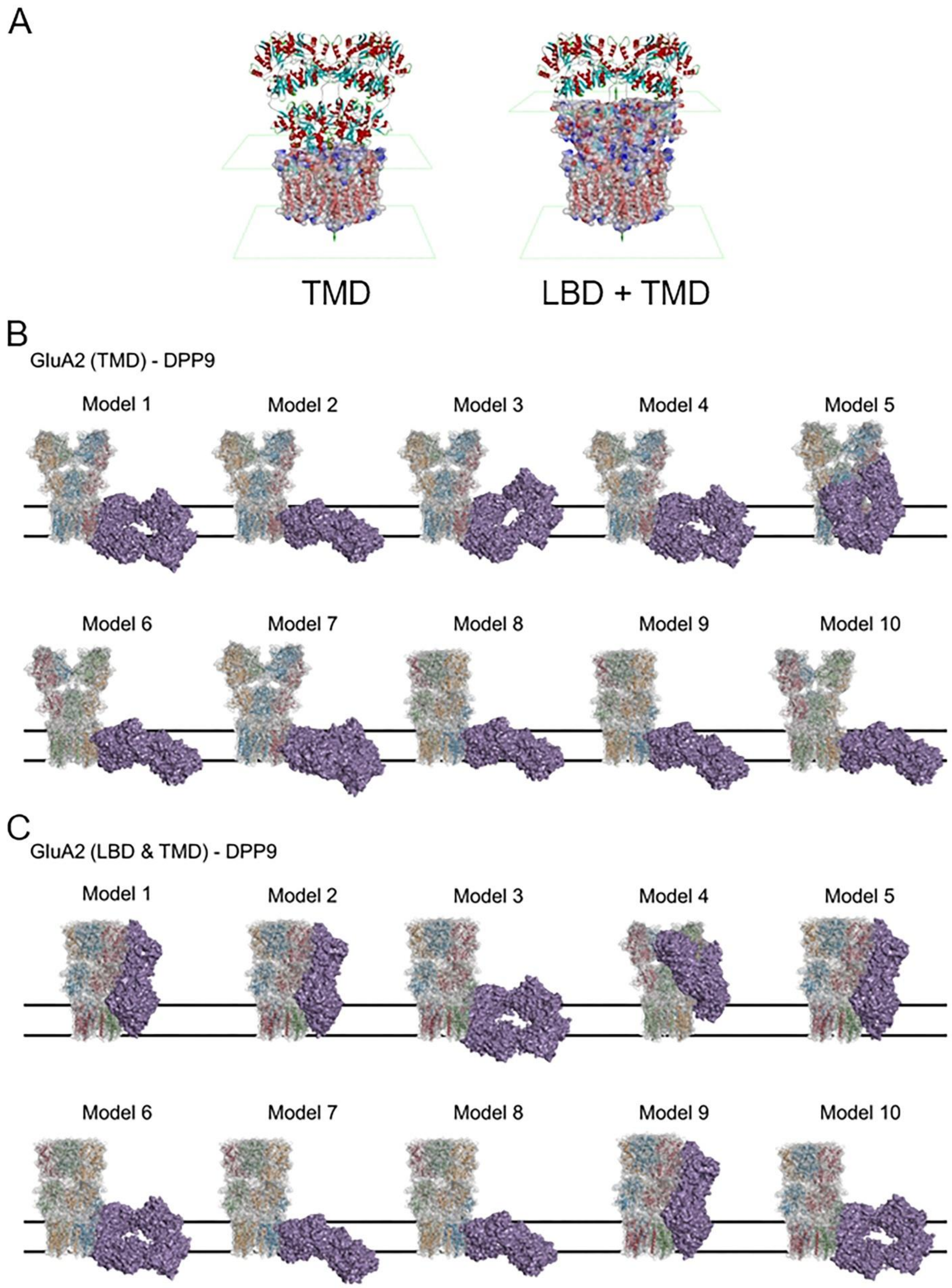

Fig. S4. GluA2-DPP9 docking simulation. **A** Schematic of two types of GluA2 protein domains for docking simulation. **B** Models for TMD of GluA2 docking with DPP9. **C** Models for LBD + TMD of GluA2 docking with DPP9.

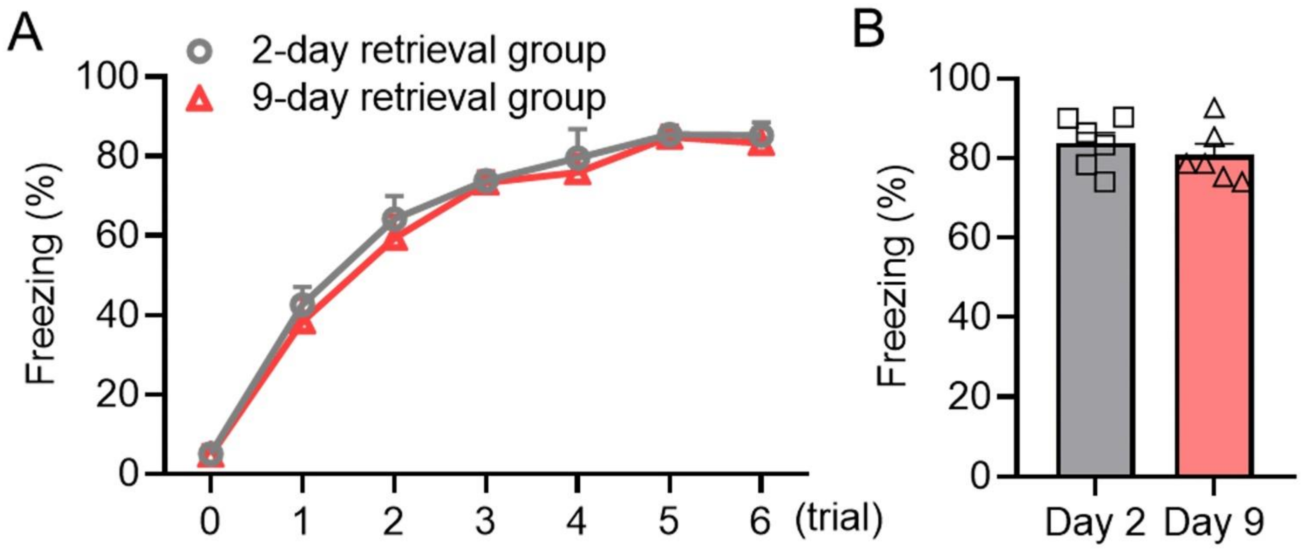

Fig. S5. The learning and retrieval performance of rats on retrieval at 2 days and 9 days post-learning. **A** Learning curves of the two groups. **B** There is no difference in memory levels between the 2-day and the 9-day retrieval.

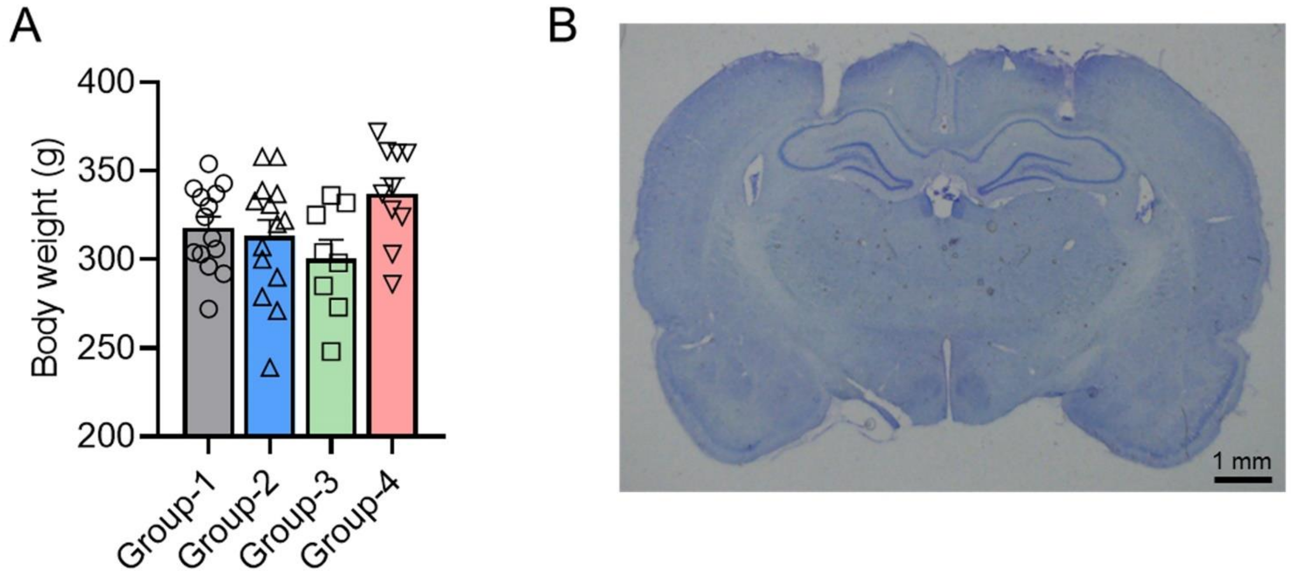

Fig. S6. Body weights and Nissl staining. **A** Body weights of the four drug groups. **B** Nissl staining for the identification of cannula location. Scale bar, 1 mm.

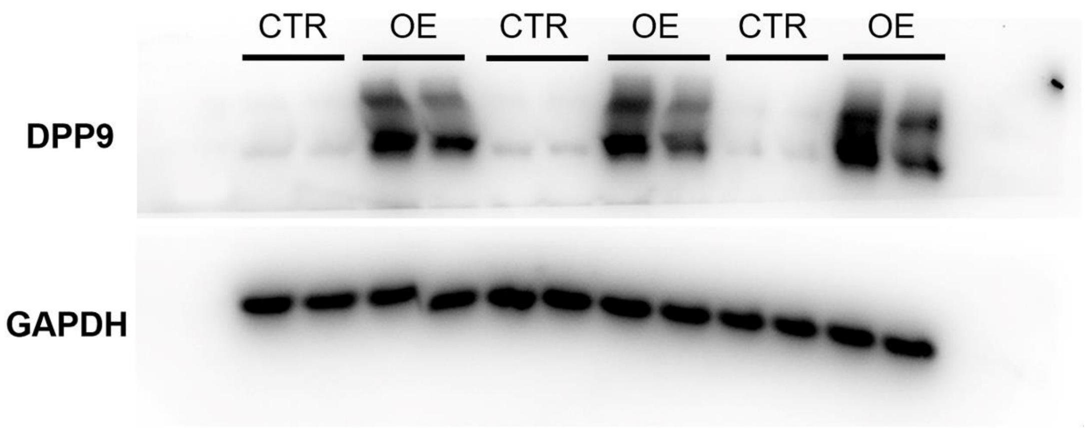

Fig. S7. Full scan of the entire original gels of Fig. 6**D**.

Table S1 Genes in cluster 49.

AC103221.1	AC121209.1	ACLY	AGFG1	ANTXR1	AQP4	ARAP2
ARMCX2	ARRDC1	ARSA	BAG1	BBS2	BRMS1	BSG
CBS	CCDC91	CCK	CD24	CHD3	CLEC2L	CMTM7
COPS4	CTSD	CYP4F17	DAD1	DBNDD2	DCAKD	DNAJC17
DNASE1L1	ECHS1	ENTPD4	ETFA	FAM129B	FAM217B	FAM222B
FCGRT	FKBP8	GAMT	GEMIN6	GFER	GKAP1	GLUL
GM6576	GPANK1	GPX4	GRIN2C	GRIP2	GTPBP2	HECW2
HSD17B10	IVNS1ABP	KCNA5	KCNJ2	KCNK2	LHFPL2	LOC100360087
LOC100360573	LOC100361457	LOC100362384	LOC100911186	LOC498555	LRRC75A	MAGT1
MOAP1	MPHOSPH6	MRPS22	MTCH1	NDUFS2	NTAN1	OPA3
PCSK7	PCYOX1	PDK1	PIBF1	PMVK	PODN	PPIL6
PPP1R8	PPP2R5A	PSMD8	PTP4A3	RAB29	RAB34	RBBP8
RERE	RFESD	RGD1559972	RPL35	RPL37A	RPS15	RPS16
RT1-CE7	SAMD10	SERINC2	SF1	SHISA5	SPINT2	TANGO2
TMEM245	TMEM258B	TMEM69	TP53INP1	TTI1	VSIR	WDR20
YIPF6	ZFP518B	ZFP566	ZFP771			

Table S2 GO enrichment of cluster 49.

**GOBP name**	***P*-value**
Vascular process in the circulatory system	0.0047
Cytosolic ribosome	0.0078
Sulfur compound metabolic process	0.0097
Ribosome	0.01
Ribosomal subunit	0.01
Carboxylic acid biosynthetic process	0.02
Organic acid biosynthetic process	0.02
Regulation of cell death	0.02
Protein localization to membrane	0.02
Protein localization to the plasma membrane	0.03
Blood circulation	0.03
Regulation of programmed cell death	0.03
Identical protein binding	0.03
Circulatory system process	0.03
